# Enhanced smart commuting with artificial intelligence for intelligent health and safety monitoring in school buses

**DOI:** 10.1038/s41598-026-41628-7

**Published:** 2026-03-21

**Authors:** Hanin Hossam, Rofida Tamer, Mariam Mohsen, Omnia Ahmed, Mennatallah Alaa, Joumana Mourad, Rana Adel, Amira Hatem, Sameh Sherif

**Affiliations:** 1 School of Computer Science and Information Technology, Egypt-Japan University of Science and Technology (E-JUST), Alexandria, Egypt; 2 School of Electronics, Communication and Computer Engineering, Egypt-Japan University of Science and Technology (E-JUST), New Borg El Arab, Egypt; 3 Biomedical Engineering Department, Capital University (formerly Helwan University), Cairo, Egypt

**Keywords:** Health care, Risk factors, Signs and symptoms

## Abstract

This paper introduces **ESC.AI (Enhanced Smart Commuting with Artificial Intelligence)**, an intelligent and integrated safety framework designed to improve health monitoring, environmental awareness, behavioral detection, driver supervision, and route optimization in school bus transportation systems. The proposed framework combines multimodal sensing, edge-based artificial intelligence, adaptive routing, and secure data management to enable proactive risk detection and real-time decision-making during transit. Although school buses remain one of the safest modes of transportation for students, recent national statistics continue to highlight persistent risks related to health emergencies, behavioral incidents, and environmental hazards. According to data from the National Safety Council (NSC) and the National Highway Traffic Safety Administration (NHTSA), school bus–related crashes resulted in 104 fatalities in the United States in 2022, representing a 3.7% decrease from 2021. Between 2013 and 2022, approximately 71% of fatalities involved occupants of other vehicles, 16% were pedestrians, and only 5% were school bus passengers. Injury statistics show a similar pattern, emphasizing the need for safety solutions that protect both students and surrounding road users. ESC.AI addresses these challenges through a unified platform that integrates Internet of Things (IoT) sensors for physiological and environmental monitoring, computer vision–based behavioral analysis, driver monitoring, and intelligent routing. Edge–cloud computing is employed to ensure low-latency responses, while blockchain-based mechanisms are used selectively to enhance data integrity, traceability, and access control for sensitive safety records. Together, these components form a cohesive and scalable framework aimed at improving transparency, responsiveness, and reliability in school transportation systems.

## Introduction

School buses are widely recognized as one of the safest modes of transportation for students; however, recent accident analyses and operational reports indicate that safety risks during transit have not been fully eliminated. Many incidents that occur during school transportation are not solely the result of traffic accidents, but are instead associated with undetected health emergencies, behavioral disturbances, or adverse environmental conditions inside the vehicle.

Existing school transportation systems typically rely on fragmented and loosely coupled solutions, where student health monitoring, behavioral supervision, environmental sensing, routing, and communication operate as isolated components. As a result, most school fleets depend heavily on manual procedures, periodic inspections, and passive surveillance methods that are not designed to provide continuous, real-time awareness. Health emergencies such as fainting or seizures may go unnoticed for critical periods, behavioral incidents such as distress or aggression are often detected late, and environmental factors including elevated CO_2_ levels, heat, and poor ventilation are rarely monitored in real time.

These limitations reduce situational awareness for drivers and administrators, delay emergency response, and weaken trust between schools and parents. Consequently, there is a clear need for an integrated and intelligent safety framework capable of continuously monitoring multiple risk factors, analyzing them in real time, and supporting timely intervention during school transportation.

### Research gap and motivation

Despite significant advances in intelligent transportation systems and smart school bus monitoring, existing solutions remain fragmented and narrowly scoped. Prior studies typically address isolated aspects of school transportation safety, such as GPS-based tracking, routing optimization, driver drowsiness detection, or student attendance monitoring. These approaches operate largely as independent modules and lack coordinated, real-time integration across health, behavior, environmental, and operational domains.

Most existing school bus safety systems rely on either cloud-centric processing or passive data logging, which limits their ability to support low-latency intervention during in-transit emergencies. Furthermore, student identification mechanisms in current deployments are predominantly based on RFID cards or optical biometrics, which are vulnerable to loss, spoofing, privacy concerns, and environmental variability. Physiological health monitoring, when present, is typically confined to clinical or laboratory settings and is rarely integrated into mobile transportation environments.

This gap is particularly critical in child-centered transportation systems, where delayed detection of health emergencies, behavioral incidents, or unsafe environmental conditions can have severe consequences. The absence of a unified, edge-enabled framework that jointly addresses physiological monitoring, behavioral intelligence, environmental awareness, secure identity verification, and adaptive routing represents a key limitation of current research and practice.

Motivated by these limitations, this work proposes ESC.AI, an integrated cyber–physical framework designed to provide continuous, real-time, and context-aware safety monitoring for school buses. By combining edge-based AI, multimodal IoT sensing, privacy-preserving biometric authentication, and safety-aware routing within a single operational platform, ESC.AI aims to bridge the gap between isolated smart transportation components and holistic, deployable school transportation safety systems.

**Contributions and Novelty** This work introduces **ESC.AI**, a school transportation safety framework that is novel in three key dimensions. First, it presents a *tissue-impedance–based biometric authentication mechanism* for student identification, leveraging intrinsic physiological electrical properties rather than image-based or token-based biometrics, thereby enhancing spoof resistance and privacy preservation. Second, ESC.AI integrates *multimodal safety sensing*, jointly combining driver behavior analysis, environmental monitoring, biometric verification, and GPS-based routing intelligence within a unified operational platform. Third, the proposed system adopts an *edge-based real-time decision-making architecture*, enabling low-latency inference and on-bus safety interventions without reliance on continuous cloud connectivity. To the authors’ knowledge, no prior school transportation system combines these three dimensions within a single, deployable intelligent framework. For example, during a routine school bus trip, a student may experience a sudden health episode such as fainting or a seizure while seated. In conventional bus monitoring systems, such incidents may remain unnoticed until the driver is alerted by other students. A real-time sensing and analytics framework could identify abnormal physiological or motion patterns earlier and trigger timely alerts for intervention.

## Problem statement

Despite maintaining relatively low crash and injury rates compared to other transportation modes, school bus systems continue to experience preventable incidents arising from systemic and technological limitations. Many of these incidents involve conditions that could be proactively detected and mitigated, including physiological health deterioration, behavioral escalation, and unsafe environmental exposure during transit.

Current school transportation solutions exhibit limited integration across sensing, analytics, and operational decision-making components. Most fleets rely on manual checklists, non-continuous health or behavioral monitoring, passive camera systems without intelligent analysis, and delayed communication with parents and school administrators. This fragmented operational structure leads to several critical deficiencies:

1. Delayed or absent detection of student health emergencies, such as fainting or seizures.
2. Inadequate identification and assessment of behavioral risks, including aggression, bullying, or psychological distress.
3. Reduced situational awareness for drivers during abnormal or emergency conditions.
4. Lack of continuous monitoring of environmental variables, such as CO_2_ concentration, temperature, and humidity, particularly during long or congested routes.

In the absence of a proactive, real-time, and multi-domain safety system, operational efficiency is reduced and emergency response effectiveness is compromised. Addressing these challenges requires a unified intelligent framework that integrates health, behavioral, environmental, and operational data into a single, coordinated platform. The **ESC.AI** model is proposed to address this gap by enabling continuous monitoring, real-time analysis, and coordinated intervention throughout the school transportation process.

## System requirements and goals

### Functional criteria

**Fig. 1:**
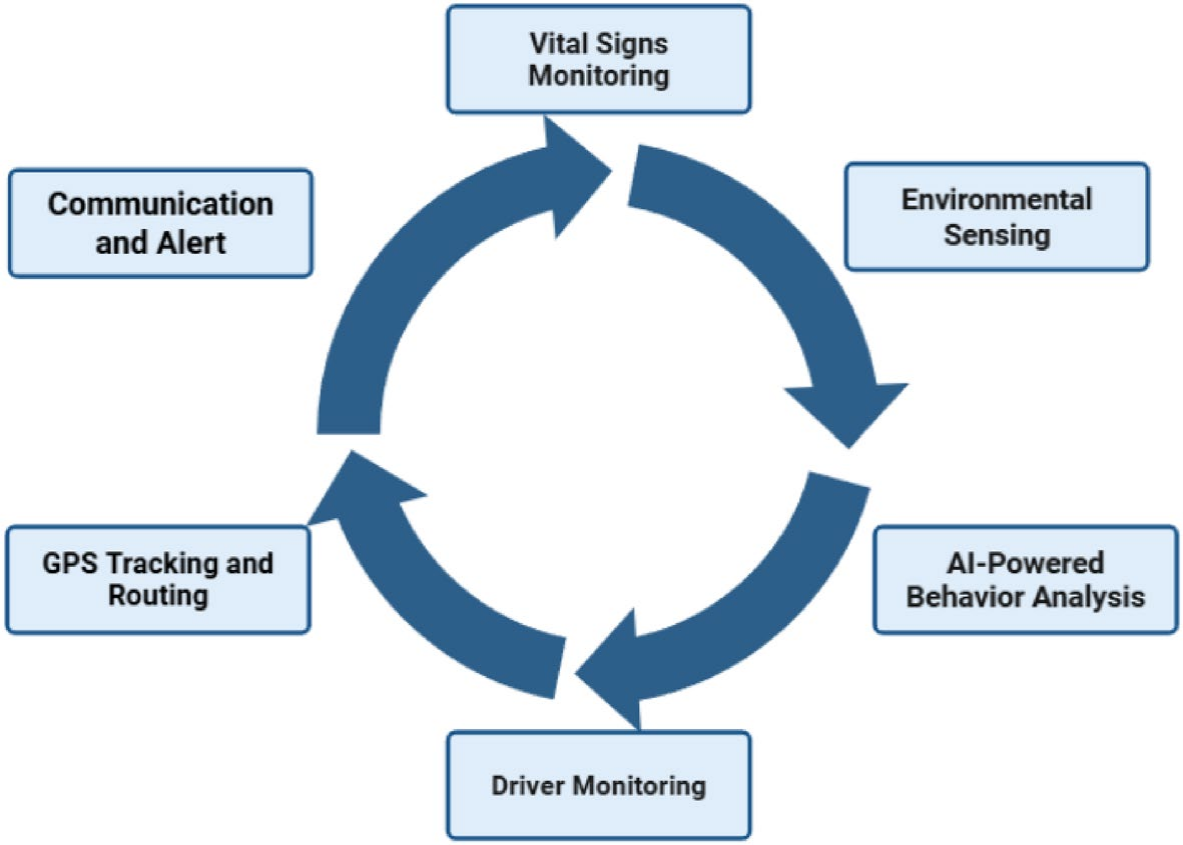
Core Functional Modules of ESC.AI System for Health, Safety, and Operational Monitoring.

To effectively address the numerous challenges of school transportation safety, the **ESC.AI** system must meet several core functional criteria. These requirements integrate IoT-based sensing, real-time AI-driven analysis, GPS tracking, and secure communication into a unified operational platform, as shown in (Fig. 1).

#### Vital signs monitoring and detection

Continuous monitoring of student health indicators, such as heart rate, body temperature, and blood oxygen saturation (SpO_2_) levels, must be supported through wearable devices that are non-invasive and safe for continuous use, or through sensors embedded within seats to minimize motion interference during measurement. Real-time algorithms must record and analyze the incoming sensor data to extract variations or abnormal parameter patterns that exceed defined thresholds, including high temperature, low oxygen levels, or irregular heart rate changes. When such abnormalities are detected, the system automatically notifies drivers and school medical personnel to enable rapid response and intervention following automated feature analysis.

#### Environmental sensing and detection

The IoT-based environmental sensing platform must continuously assess temperature, humidity, CO_2_ concentration, and other air quality parameters. Elevated CO_2_ levels are known to impair concentration and induce drowsiness, while excessive heat or poor ventilation increases the risk of dehydration and discomfort. When environmental conditions exceed safety limits, the system must immediately trigger real-time alerts and provide the driver with actionable recommendations to restore safe cabin conditions.

#### AI-based behavior analysis

Camera-based behavioral monitoring must utilize advanced computer vision techniques to detect aggression, distress, falls, or unsafe student actions. The AI must be trained on diverse datasets to ensure robustness under varying lighting and seating arrangements. Detected incidents should be timestamped, logged, and optionally transmitted to school administrators to ensure accountability and improve student safety.

#### Driver Monitoring System (DMS)

The Driver Monitoring System evaluates driver attentiveness and behavioral state using in-cabin vision sensors. The system is designed to detect indicators of fatigue, distraction, mobile phone usage, and seatbelt non-compliance. Upon identification of a risky behavior, the system provides real-time alerts to the driver and records the event for subsequent review.

To support real-time inference on resource-constrained edge devices, the DMS utilizes the YOLOv8 object detection framework^1^, selected for its balance between detection accuracy and computational efficiency. The model is trained using a publicly available driver monitoring dataset containing diverse lighting conditions, head poses, and occlusion scenarios. Data augmentation techniques, including brightness variation, random cropping, and horizontal flipping, are applied to improve generalization performance.

**Dataset Description:** The model was trained and improved using the YOLOv8 driver monitoring dataset^2^, which has thousands of labeled photos that show how people drive in the actual world. There are different lighting, occlusion, head position, and face expressions in the dataset to make sure it can be used in many different situations. To improve performance in different environments, we used data augmentation methods including random cropping, brightness scaling, and horizontal flipping.

#### GPS tracking and intelligent routing

The system incorporates continuous GPS-based vehicle tracking to enable real-time monitoring of bus locations by parents and school administrators. Routing decisions are informed by dynamic inputs such as traffic congestion, weather conditions, school zone constraints, and road safety indicators. The routing module supports adaptive reconfiguration in response to unexpected delays or emergency situations to enhance reliability and safety throughout the journey.

#### Communication and alert system

ESC.AI employs a multi-channel communication framework to disseminate alerts related to health conditions, behavioral incidents, environmental anomalies, and routing updates. Notifications are delivered through SMS, mobile applications, and web-based dashboards to ensure timely awareness and coordination among drivers, school staff, and parents.

### Goals and objectives

**Fig. 2:**
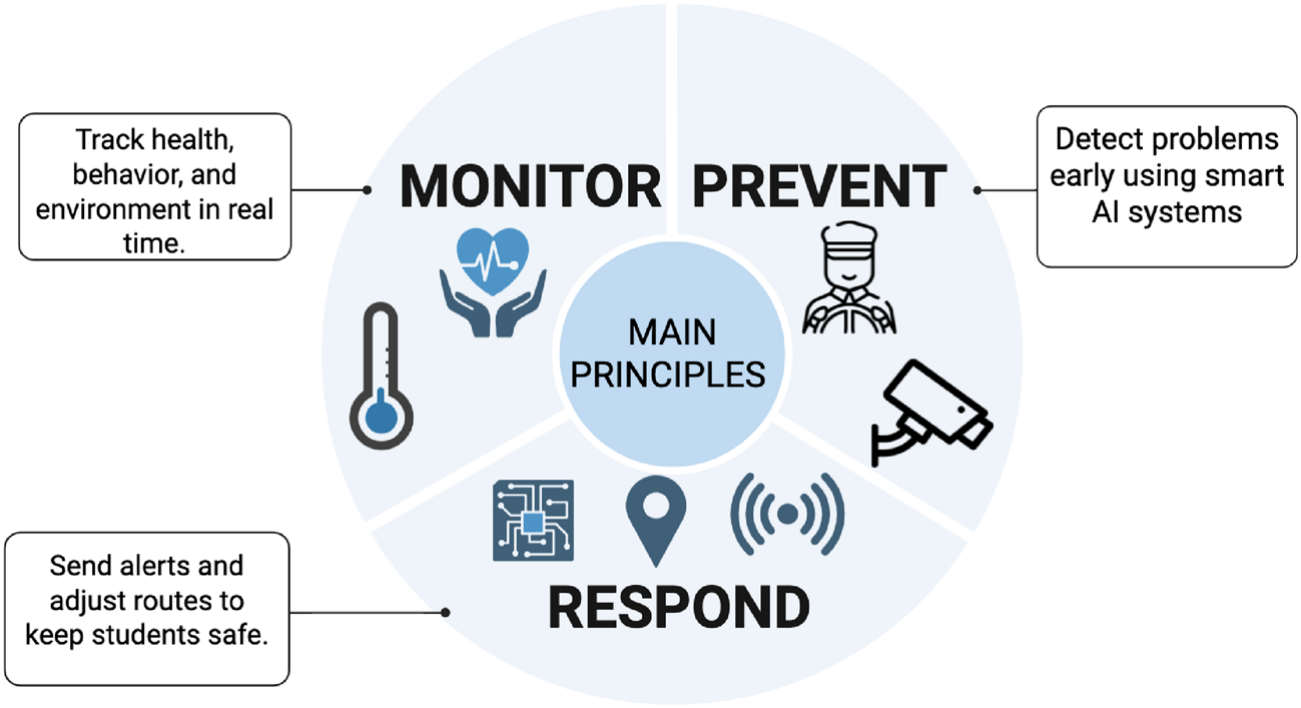
ESC.AI Core Operational Principles: Monitor, Prevent, and Respond.

The primary objective of **ESC.AI** is to modernize school transportation safety by integrating intelligent monitoring and decision-support technologies into a cohesive framework, as illustrated in Fig. 2. The system goals are defined as follows:

#### Goal 1: Health monitoring and emergency identification

Enable continuous monitoring of student physiological and environmental conditions to support early detection of health emergencies and timely response.

#### Goal 2: Behavioral and driver safety monitoring

Improve student and driver safety by identifying risky behaviors and unsafe conditions using vision-based and sensor-driven analysis.

#### Goal 3: Secure and compliant data management

Ensure the integrity, traceability, and controlled access of sensitive transportation data through secure storage, encryption, and privacy-aware mechanisms.

#### Goal 4: Adaptive and safety-aware route optimization

Enhance route efficiency and reliability through dynamic routing strategies that account for traffic conditions, environmental factors, and safety constraints.

#### Goal 5: Effective communication and coordination

Provide timely, transparent, and reliable communication among all stakeholders during routine operations and emergency situations.

#### Goal 6: Privacy and cybersecurity assurance

Protect all collected data through robust cybersecurity practices, access control policies, and compliance with relevant privacy regulations.

#### Goal 7: Scalability and real-time operation

Support scalable deployment across multiple vehicles and routes through edge-based processing and cloud-assisted coordination while maintaining real-time responsiveness.

**ESC.AI** introduces a unified, intelligent approach to school transportation safety by integrating health monitoring, behavioral analysis, environmental sensing, driver assessment, and adaptive routing into a single framework. By combining AI, IoT, and advanced communication technologies, the system establishes a foundation for next-generation smart mobility solutions that protect students, support institutions, and empower families through transparency and real-time decision-making.

## Related work

A significant amount of research has investigated the enhancement of school bus routing via computational and algorithmic methodologies, encompassing metaheuristic approaches, real-time GPS applications, and traffic-sensitive decision frameworks. Traditional formulations of the Vehicle Routing Problem (VRP) have been extensively examined within the realm of school transportation, establishing the basis for several routing techniques^3^. More recent work has employed artificial intelligence and machine learning to support dynamic routing decisions under changing conditions^4,5^. Despite these advances, many traditional optimization methods rely on static constraints and assumed environmental certainty. Consequently, such methods often underperform in real-world school transportation systems characterized by unpredictable traffic delays, safety concerns, and strict time constraints. The advancement of machine learning techniques has improved routing flexibility under dynamic conditions. Deep learning models trained on historical traffic patterns have demonstrated the ability to predict congestion and recommend alternative routes, thereby improving operational efficiency^6^. Complementary IoT-based systems collect sensor data from urban infrastructures to support intelligent planning for public and school transportation networks^7^. However, many of these systems lack multi-contextual awareness, particularly real-time behavioral integration and explicit linkage to student safety practices, which are critical in child-centered transportation environments. Ant Colony Optimization (ACO), a swarm intelligence–based technique, has shown strong potential for route optimization. A foundational study^8^ demonstrated that ACO can reduce travel distance and improve routing efficiency. A study^9^ further applied ACO to school bus routing using dynamic optimization strategies. Despite these contributions, many ACO-based approaches remain limited to offline simulations and do not incorporate real-time GPS data or adaptive rerouting based on environmental changes. Hybrid metaheuristic approaches, such as those combining genetic algorithms^10^, improve convergence speed but often introduce substantial computational overhead, limiting their suitability for deployment on resource-constrained edge devices commonly used in school buses. Safety-aware routing has also received increased attention. Various studies^11^ proposed integrating crime statistics and environmental so consider a vital risk factors into routing strategies to enhance student safety. Other studies have explored AI-enhanced mechanisms capable of recognizing and responding to hazards in real time. Nevertheless, these works typically treat routing optimization, safety monitoring, and behavioral analysis as independent subsystems rather than components of a unified decision-support framework. Related studies have explored student tracking and identification. Different studies^12^ developed an IoT-based system utilizing ESP32, GPS, RFID^13,14^, and cloud-based services to track student boarding and bus location. While effective for attendance tracking and notifications, the system does not incorporate physiological health monitoring, behavioral analysis, or edge-level intelligent decision-making. Recent research increasingly integrates biometric identification with AI-driven analytics to enhance student monitoring capabilities. Alfaifi *et al.*^15^proposed a comprehensive safety framework addressing unattended minors, overcrowding, driver irregularities, and pedestrian protection using RFID-based bracelets. However, their approach does not integrate physiological sensing, computer vision–based analysis, or secure data management mechanisms. Biometric identification technologies have also advanced through artificial intelligence integration. Grosz et al^16^. introduced AFR-Net, a fingerprint-based architecture that improves matching accuracy. While effective for identity verification, such approaches operate independently of behavioral, environmental, and health monitoring, limiting their applicability to holistic student safety management. Driver monitoring has likewise been widely studied. Rupani *et al.*^17^ developed a real-time drowsiness detection system using eye aspect ratio and facial landmarks on embedded platforms. More recent AI-powered systems extend these capabilities to detect distraction, unsafe behavior, and compliance with safety regulations. Overall, existing research addresses individual aspects of school transportation safety, including routing optimization, student tracking, biometric identification, environmental sensing, and driver monitoring. However, there remains a lack of a unified, AI-driven, real-time operational framework that integrates these components into a single decision-support system capable of jointly addressing physiological health, behavioral intelligence, environmental awareness, secure identity management, and adaptive routing. expand more

## Datasets and experimental setup

This section describes the datasets employed to develop and evaluate the proposed ESC.AI framework. Multiple heterogeneous datasets were utilized to support vision-based safety monitoring, physiological health assessment, audio-based respiratory analysis, and biometric identity verification. All datasets were selected or collected to reflect realistic operating conditions in school transportation environments and to support reproducible experimental evaluation.

### Driver monitoring dataset

A custom image dataset was constructed to train and evaluate the driver monitoring subsystem. The dataset consists of annotated in-cabin images capturing five safety-critical driver behaviors: *Open Eye*, *Closed Eye*, *Cigarette*, *Phone*, and *Seatbelt*. Images were organized into mutually exclusive training, validation, and testing subsets following the standard YOLOv8 directory structure. Manual bounding-box annotations were provided for all object classes to enable supervised object detection. This dataset supports real-time detection of driver distraction, fatigue, and safety compliance under varying illumination and in-cabin conditions.

### Bus violence dataset

To evaluate in-vehicle violence detection, the Bus Violence Dataset was employed. This benchmark dataset contains 1,400 short video clips recorded inside a moving bus using three synchronized cameras operating at 25 frames per second. The dataset is perfectly balanced, comprising 700 violent and 700 non-violent clips, with clip lengths ranging from 16 to 48 frames. Videos capture a wide range of passenger interactions, including simulated physical aggression and non-violent behaviors. The dataset was used for both training and testing spatio-temporal deep learning models, enabling robust evaluation of real-time violence detection in realistic public transport scenarios.

### Seizure detection dataset

Seizure detection experiments were conducted using EMG components extracted from the CHB-MIT EEG dataset. The dataset consists of multi-channel physiological recordings sampled at 256 Hz and annotated for seizure events. EMG-related signal components were segmented into fixed-length windows and labeled based on seizure presence within each segment. Preprocessing included bandpass filtering, normalization, and temporal segmentation. Both classical machine learning models and deep learning architectures were evaluated, with CNN–LSTM serving as the primary model and Random Forest and SVM used as baseline comparators.

### Respiratory sound dataset

Respiratory health assessment was performed using the publicly available Respiratory Sound Database collected by research teams in Portugal and Greece. The dataset includes 920 audio recordings from 126 patients, totaling approximately 5.5 hours of respiratory sounds and 6,898 annotated respiratory cycles. Each cycle was labeled as *Normal*, *Wheeze*, or *Crackle*. Analysis was conducted at the respiratory cycle level to support fine-grained detection of abnormal breathing patterns. Audio preprocessing included noise reduction, segmentation, MFCC feature extraction, and normalization. Deep learning models (CNN–GRU and LSTM–CNN) were employed, with traditional classifiers such as SVM used for baseline comparison.

### ECG heartbeat dataset

ECG-based cardiac monitoring experiments utilized recordings from two well-established sources: the MIT-BIH Arrhythmia Database and the PTB Diagnostic ECG Database. The MIT-BIH dataset provides annotated single-lead ECG recordings sampled at 360 Hz, while the PTB dataset includes high-resolution multi-lead ECG signals sampled at 1000 Hz. To ensure consistency, all ECG signals were downsampled to 125 Hz, segmented into fixed-length windows, and normalized. Classification was performed at the window level to enable continuous monitoring. A CNN–LSTM architecture was selected as the primary model for detecting stress-related cardiac irregularities, with CNN and GRU models included for comparative evaluation.

### Biometric impedance dataset

A custom biometric impedance dataset was collected to evaluate tissue-impedance-based identity verification. The dataset comprises impedance spectra acquired from 20 healthy volunteers, with measurements collected from five fingers per subject across multiple sessions. Interdigitated microelectrode sensors were used to record complex impedance responses over an excitation frequency range of 50 Hz to 5 MHz. Ratiometric impedance features were extracted to mitigate sensitivity to contact pressure and environmental variation. Samples were labeled at the finger level and evaluated using subject-independent cross-validation. The dataset was designed to reflect realistic deployment conditions, supporting robust biometric authentication in mobile and resource-constrained transportation environments.

## Methodology

### Overview of ESC.AI framework

**Fig. 3:**
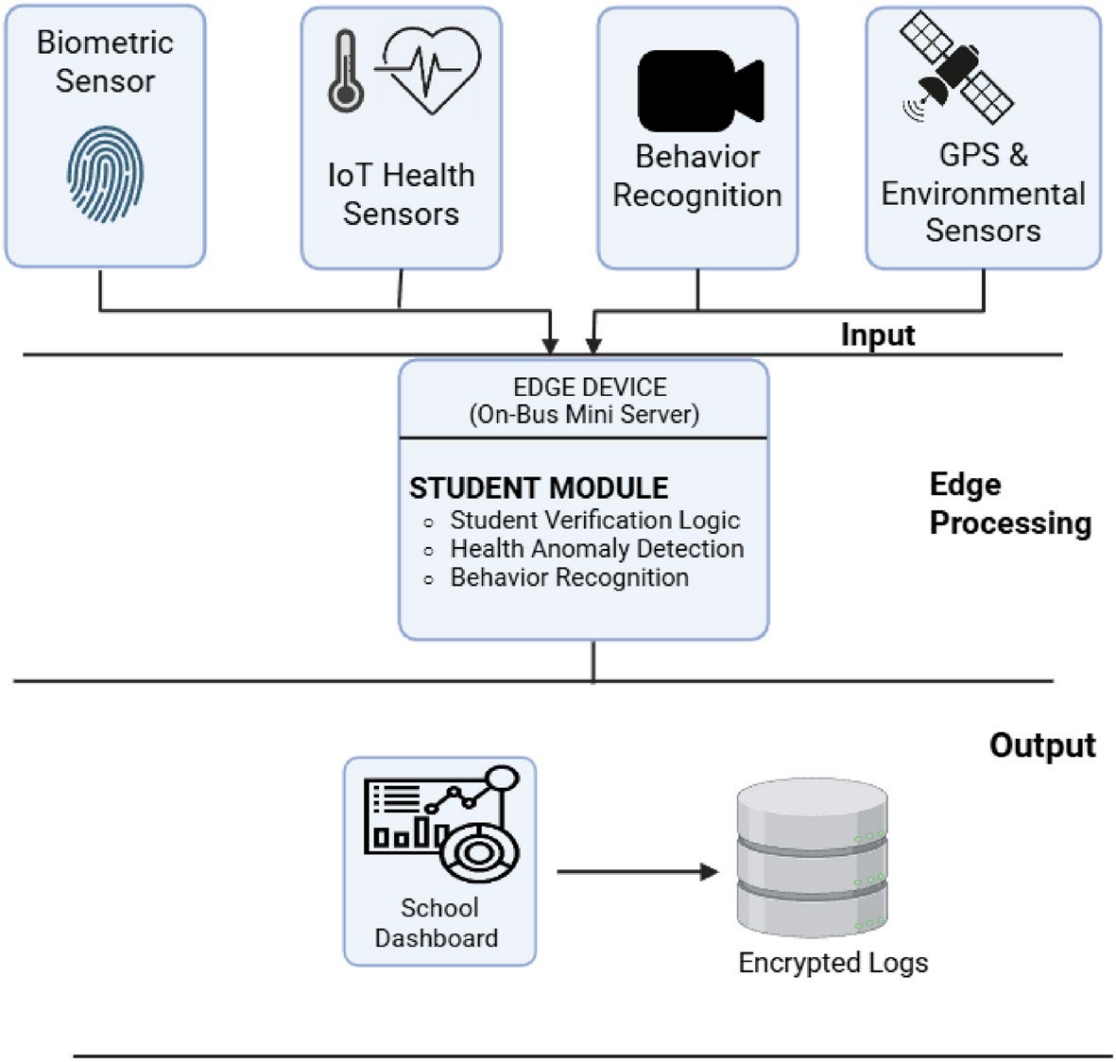
ESC.AI System Architecture illustrating integrated sensing, behavioral analysis, routing optimization, and communication layers.

ESC.AI is a complete, flexible, and cyber-physical platform that offers smart health, safety, and operational monitoring for school bus transportation systems (Fig. 3). Unlike typical fragmented systems, ESC.AI combines neuro-physiological sensors (ECG, EEG, EMG), adaptive routing algorithms^18^, and in-cabin behavioral AI into a single architecture that works well in mobile, low-resource settings^7,19–21^. The main parts of ESC.AI are a network of wearable biosensors, embedded microelectronics, AI-enabled cameras, edge computing modules, and communication interfaces based on the Internet of Things (IoT).

Physiological monitoring is facilitated by seat-integrated or wearable sensors that gather continuous signals, such as electrocardiography (ECG) for cardiac activity, electroencephalography (EEG) for neural states, and electromyography (EMG) for muscular activation, alongside environmental indicators like CO_2_ concentration, temperature, and humidity that have a direct effect on the safety and comfort of students^22^. To make sure that health signals are analyzed quickly, bus-mounted edge computing units run lightweight deep-learning models that can find abnormal heart rhythms, breathing problems, muscle inactivity, or other problems in real time^22^. Edge processing cuts down on latency, lets devices work on their own when the internet is down, and makes them less dependent on cloud infrastructure?.

At the same time, computer vision models based on YOLOv8 look at live video feeds from in-cabin cameras to find dangerous driver behaviors such as being distracted, sleepy, using a cell phone, or not wearing a seatbelt. They also look for student-related dangers like falls, violence, or discomfort. ESC.AI uses embedded visual intelligence to do real-time risk assessments, provide alerts right away, and report incidents to the cloud. This is different from passive surveillance systems.

An improved Ant Colony Optimization (ACO) framework^9^ was created to solve the School Bus Routing Problem (SBRP) under dynamically changing constraints. The framework employs pheromone-driven mechanisms to adapt route selection in real time based on geospatial traffic data, crime density statistics, weather conditions, and road safety indicators^10,11^. This dynamic routing strategy is intended to reduce travel distance and delay while prioritizing safer transit corridors^23^. By incorporating safety-aware routing decisions, the system aims to improve passenger safety^5^ and operational efficiency, including potential reductions in fuel consumption^24^. The ability to reroute dynamically supports timely responses to traffic disruptions or health-related events, contributing to overall system robustness.

ESC.AI combines perceptual sensing, AI-driven analytics, and adaptive routing to enhance situational awareness within school transportation systems. By integrating real-time monitoring and decision-support capabilities, the framework is designed to support safer operation, improved reliability, and timely response to abnormal or emergency conditions in modern school bus environments^7,25^.

### System components and layered architecture

**Fig. 4:**
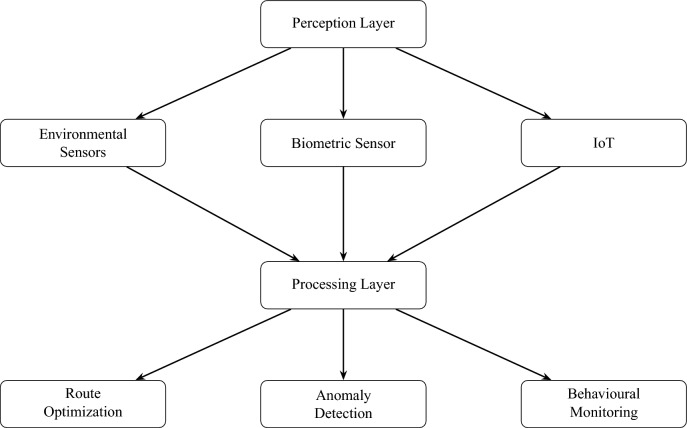
Layered system architecture of ESC.AI illustrating perception, processing, and routing-related components.

The **ESC.AI** architecture (Fig. 4) is composed of four tightly integrated layers, each responsible for a distinct operational function ranging from data acquisition to real-time AI inference, secure communication, and decision support for stakeholders^26^. This layered and modular design supports scalability, adaptability, and resilient operation across diverse deployment environments.

#### Perception layer — data acquisition

The perception layer serves as the data acquisition component of **ESC.AI**, enabling continuous collection of physiological, environmental, behavioral, and locational information. By integrating heterogeneous sensors, each school bus functions as a mobile sensing platform capable of supporting real-time situational awareness^15,27^.

The proposed system incorporates multiple physiological sensors to monitor student health conditions. ECG sensors, such as the AD8232, analyze cardiac electrical activity to detect arrhythmias and derive stress-related metrics, including heart rate variability (HRV)^19^. EMG sensors^28^ capture muscular activity that may indicate fatigue, tremors, or prolonged inactivity. This multimodal physiological sensing approach supports continuous health monitoring and facilitates early identification of potential abnormalities.

The system also integrates environmental and auxiliary sensors to support safe transportation conditions. MH-Z19B CO_2_ sensors continuously monitor ventilation quality to mitigate drowsiness and cognitive impairment^22,29^. Temperature and humidity sensors, such as the DHT22, track thermal comfort levels that may affect student well-being. High-definition cameras function as vision sensors, capturing continuous video streams of student and driver activity. AI-based detection models, including YOLOv8^1^, analyze these streams in real time to identify potentially unsafe events. GPS modules, such as the NEO-6M, provide geolocation data for route planning, arrival time estimation, and off-route detection.

Additional safety measures include alcohol detection sensors (MQ-3) to assess driver sobriety before and during operation^30^. Biometric verification is supported through fingerprint scanners based on Electric Impedance Spectroscopy (EIS)^31^, enabling reliable identity verification during boarding and departure events and improving attendance accuracy and security logging.

The integration of heterogeneous sensing modalities enables contextual interpretation of physiological status, environmental conditions, behavioral patterns, and geospatial information in real time. This multimodal perception layer provides the foundational data required for downstream analysis and decision-support functions within the **ESC.AI** framework.

#### Processing layer — edge intelligence and AI inference

These models include YOLOv8 for vision-based behavior detection^32^, CNN–LSTM architectures for temporal analysis of physiological signals such as ECG^19^and EMG^33^, and MFCC–GRU networks for respiratory sound classification^21^. The CNN components are responsible for spatial feature extraction from raw signal representations, while recurrent units (LSTM and GRU) capture temporal dependencies critical for identifying abnormal patterns over time. Model selection prioritizes lightweight architectures suitable for edge deployment with constrained computational resources. This edge-centric architecture reduces latency and supports continuous monitoring even in connectivity-limited environments^15^. All AI models are trained offline and deployed exclusively for inference, enabling efficient and reliable real-time operation.

**b)** The signal processing pipeline is designed to ensure robust feature extraction prior to AI inference. Incoming biosignals, including ECG and EMG, undergo noise filtering and peak detection before extraction of key features such as heart rate variability (HRV) and root mean square (RMS) values. Audio signals are processed using Mel-Frequency Cepstral Coefficients (MFCCs) to extract discriminative spectral features for respiratory sound classification. Normalization procedures are applied across signal modalities to reduce inter-subject variability and improve model generalization. The extracted features are formatted into fixed-length temporal windows and forwarded to the corresponding AI inference modules, ensuring compatibility with recurrent model inputs and consistent real-time operation across sensing modalities.

**Fig. 5:**
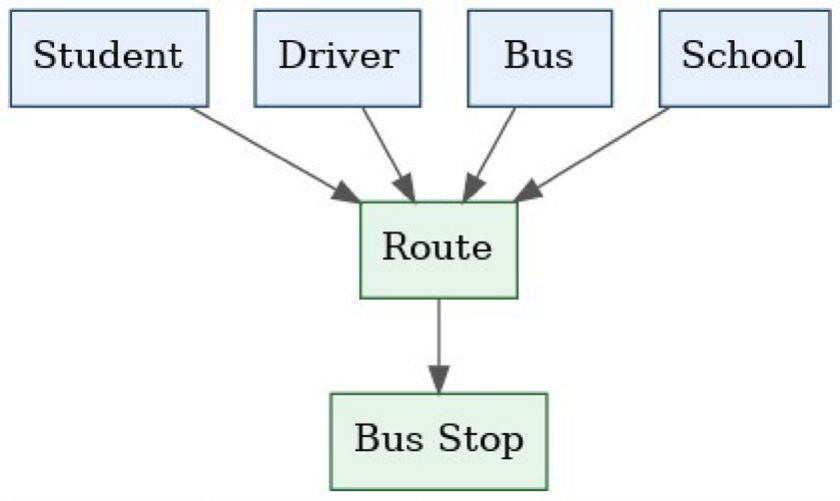
Conceptual representation of multi-stakeholder interaction in school transportation routing, highlighting the need for a shared and verifiable trust layer.

**Fig. 6:**
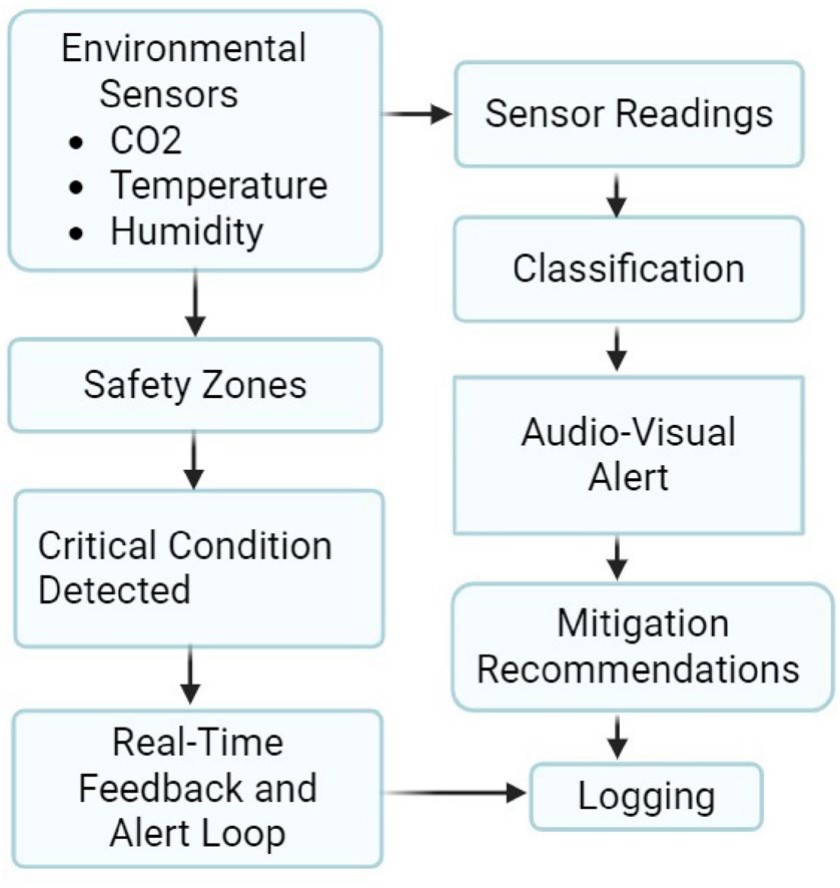
Environmental Monitoring and Feedback Loop in ESC.AI.

**c)** The Driver Monitoring System (DMS) within the **ESC.AI** framework employs the YOLOv8 object detection architecture^34^, a one-stage detector optimized for low-latency inference. YOLOv8 processes input video frames to simultaneously perform object localization and classification, enabling efficient detection of driver-related behaviors in real time^34^. The model operates on resized RGB and infrared (IR) image streams to balance detection accuracy with computational efficiency on embedded edge devices. The DMS is designed to detect multiple categories of risky driving behavior (Fig. 5), including seatbelt non-compliance, smoking, mobile phone usage, and prolonged eye closure indicative of drowsiness. The detection and classification workflow is illustrated in Fig. 6. Each identified event is time-stamped, assigned a confidence score, and forwarded to the real-time alerting and logging modules to support prompt intervention and longitudinal safety analysis^15^.

Ensuring driver readiness is critical in child-centered transportation environments. The Driver State Monitoring Module in **ESC.AI** follows a supervisory design inspired by established DMS frameworks^35^. It integrates computer vision–based analytics^36^, biometric verification mechanisms^37^, and behavioral risk assessment models to monitor alertness and detect potentially unsafe actions. Unlike conventional telematics systems that react post-incident, the proposed framework emphasizes early risk detection to support preventive safety interventions. The modular layered architecture of ESC.AI enables horizontal scaling by allowing independent deployment and extension of perception, processing, and application components as system load increases.

#### Alert recommendation mechanism

The alert recommendation mechanism in ESC.AI is designed as a rule-assisted intelligent decision layer that translates monitored events into actionable alerts. Rather than relying solely on fully autonomous decision-making, the mechanism follows a hybrid approach that combines predefined safety rules with AI-assisted confidence scoring.

At the initial stage, threshold-based rules are applied to continuously monitored signals, such as abnormal physiological readings, prolonged inactivity, route deviation, or sensor malfunction. When a predefined condition is violated, a candidate alert is generated and assigned a priority level (low, medium, or high) based on event severity and contextual factors.

For selected alert categories, AI-based models are designed to assist in refining alert confidence by analyzing temporal patterns and historical context. However, these AI-driven refinements are presented as future extensions of the framework and are not fully deployed in the current implementation. Alert dissemination is performed in real time to relevant stakeholders, including drivers, school administrators, and parents, depending on alert type and severity.

#### AI/ML techniques and deployment

ESC.AI employs a hybrid AI architecture integrating rule-based logic, classical machine learning, and deep learning models:

- **Rule-based**: Threshold-based alert generation for physiological and environmental signals.
- **Classical ML**: Random Forest and XGBoost classifiers for tissue-impedance biometric authentication. Achieved classification accuracies of 91–92%, with precision, recall, and F1-scores exceeding 90%.
- **Deep Learning**: YOLOv8 for driver and student behavioral monitoring, CNN-LSTM for temporal analysis of ECG and EMG, and MFCC-GRU networks for respiratory sound classification.

All AI inference is performed on edge devices to ensure low-latency, real-time operation, while model training is conducted offline in cloud environments. Performance metrics reported reflect experimental evaluation where available. AI-assisted extensions for enhanced alert confidence and cross-module predictive analytics are planned as future work. Table 1 summarizes the AI/ML techniques employed in ESC.AI, along with their execution location and design justification.

**Table 1:** AI/ML Components in ESC.AI: Technique, Execution Location, and Justification.

Component	Technique type	Specific method	Device	Location	Justification
Environmental Threshold Alerts	Rule-Based	Deterministic comparisons	Edge	On-bus	Guaranteed response; no training required
Driver Behavior Detection	Deep Learning	YOLOv8 CNN	Edge	On-bus	State-of-the-art accuracy; single-pass inference
Arrhythmia Classification	Deep Learning	1D CNN	Edge	On-bus	Temporal pattern recognition in ECG
Respiratory Sound Analysis	Deep Learning	CNN-LSTM hybrid	Edge	On-bus	Combined spatial-temporal features
Stress Detection	Deep Learning	GRU	Edge	On-bus	Temporal HRV pattern recognition
Seizure Detection	Classical ML	Random Forest	Edge	On-bus	Interpretable; low computational cost
Violence Detection	Deep Learning	I3D ConvNet	Edge	On-bus	Spatio-temporal video analysis
Fall Detection	Deep Learning	LSTM	Edge	On-bus	Temporal pose sequence analysis
Environmental Risk Classification	Classical ML	Decision Tree	Edge	On-bus	Interpretable; explainable decisions
Hypertension Risk Prediction	Classical ML	Random Forest	Edge	On-bus	Multi-feature classification
Route Optimization	Metaheuristic	Ant Colony Optimization	Cloud	Off-bus	Requires global fleet coordination
Model Training/Retraining	Deep Learning	Various	Cloud	Off-bus	Computationally intensive; offline

#### Storage and communication layer

The **Storage and Communication Layer** is the part of the **ESC.AI** system that manages data. It makes sure that there is safe preservation, organized transmission, and limited access to AI-generated insights and multimodal sensor streams. Acting as a bridge between the edge-processing units on board and the decision platforms in the cloud, this layer supports real-time reactivity while ensuring that data is reliable, traceable, and stays safe for a long time.

**Real-Time Alerting System:** The ESC.AI framework incorporates a real-time alerting system designed to support timely and context-aware safety interventions during school transportation. Alerts are generated locally at the edge based on continuous analysis of multimodal sensor data, ensuring low-latency response without reliance on persistent cloud connectivity.

Alert triggers originate from three primary sources: (i) environmental sensing, including cabin CO_2_ levels, temperature, and humidity; (ii) physiological and biometric signals related to student or driver health conditions; and (iii) motion- or behavior-related anomalies detected through embedded sensors. Incoming data streams are evaluated against predefined safety thresholds derived from operational guidelines.

To manage urgency and response, alerts are classified into three priority levels: *informational*, *warning*, and *critical*. Informational alerts indicate non-critical deviations, such as gradually increasing CO_2_ levels, and typically recommend corrective actions such as improving ventilation. Warning alerts correspond to abnormal patterns that may pose safety risks if unaddressed, including early indications of physiological distress. Critical alerts are triggered by severe or abrupt anomalies, such as suspected health emergencies, and prompt immediate driver notification along with escalation to designated school authorities or emergency contacts.

Alert notifications are delivered through multiple channels, including SMS messages, push notifications, and dashboard warnings. This rule-based alert generation and recommendation mechanism prioritizes transparency, reliability, and interpretability in safety-critical scenarios, while more advanced adaptive alert strategies are considered as part of future system enhancements.

**Parent Portal Application:** A specialized mobile application provides parents and guardians with continuous access to transportation information. Features include real-time GPS tracking, estimated time of arrival (ETA), health status updates, automated boarding and departure verification, and secure two-way communication with school transportation staff. This enhances transparency and strengthens coordination between parents and school authorities.

**Central Dashboard Interface:** School administrators use a centralized web-based dashboard to monitor system-wide data streams, including vehicle telemetry, health indicators, behavioral analytics, and route progress. The dashboard supports incident logging, compliance monitoring, historical trend analysis, and long-term operational planning.

**Secure Data Transmission:** All communications between vehicle-mounted edge units, cloud servers, and user interfaces are protected using AES- and RSA-based encryption techniques. Session-based authentication and offline buffering mechanisms ensure data security and system continuity, even under intermittent or degraded network conditions^38^.

#### Application layer

The application layer provides visualization, decision-support, and operational management tools that transform raw and processed data into actionable insights.

**Live Monitoring Dashboards:** Live dashboards allow school officials and fleet operators to monitor buses in real time, displaying health alerts, behavioral indicators, and environmental conditions for each vehicle through intuitive visual representations.

**Route Optimization Interface:** The ACO-based routing engine generates optimized route recommendations that are presented through an interactive interface. The system supports dynamic route adaptation in response to traffic conditions, detected risks, or emergency events^9^.

### Role of blockchain in the proposed school transportation safety system

In the proposed ESC.AI framework, blockchain is utilized as a decentralized trust and integrity layer rather than a full-scale transactional platform. The primary objective of incorporating blockchain is to ensure immutability, traceability, and auditability of safety-critical transportation events generated during school bus operations.

School transportation systems involve multiple independent stakeholders, including students, drivers, schools, parents, and regulatory authorities. In such multi-actor environments, reliance on a centralized database may raise concerns related to data tampering, unauthorized modification, and limited transparency. To address these challenges, blockchain is conceptually introduced as a shared, tamper-resistant ledger that enables independent verification of critical safety events without dependence on a single trusted authority.

To minimize storage overhead and preserve data privacy, the system does not store raw sensor data or personally identifiable information on-chain. Instead, the blockchain records only:

- Cryptographic hashes of safety-related events (e.g., student boarding confirmation, route deviation alerts, emergency incidents),
- Timestamped transaction identifiers,
- Event verification and validation status.

All detailed sensor readings, physiological signals, and video data are securely stored off-chain, while blockchain entries serve as integrity proofs that allow post-incident verification and forensic auditing. This hybrid on-chain/off-chain design ensures data authenticity while maintaining scalability and compliance with privacy requirements.

Compared to traditional secure centralized databases, blockchain-based logging provides several advantages, including immutability that prevents retroactive data modification, decentralized verification that reduces single-point-of-failure risks, and enhanced auditability that supports transparent post-incident investigations. These properties are particularly valuable in safety-critical and child-centered transportation systems, where trust, accountability, and data integrity are paramount.

It is important to note that the blockchain component in this work is conceptual and architectural in nature. The focus of the study is on system design and security modeling rather than platform-specific blockchain deployment. Performance evaluation, smart contract implementation, and real-world blockchain integration are identified as directions for future research.

### Scope and limitations

The proposed ESC.AI framework focuses on architectural design, system integration, and proof-of-concept evaluation of selected AI components. While several AI-based modules are experimentally validated using public and custom datasets, other intelligent functionalities are presented at a conceptual level and are intended for future implementation and large-scale validation. This scope allows the framework to remain flexible while highlighting promising research directions. Scalability testing under large-scale deployment scenarios, such as city-wide school transportation systems with hundreds of buses and thousands of students, is beyond the scope of the current study. The proposed architecture is designed with modular and distributed components that are intended to support future scalability evaluation and optimization.

## Smart bus intelligence and optimization

### Environmental monitoring and air quality management

Maintaining optimal in-cabin environmental conditions (Fig. 6) in school buses is critical not only for comfort but also for safeguarding student health, cognitive performance, and overall safety during transit. Prior studies by Satish *et al.*^22^ and Mendell *et al.*^29^ demonstrate that elevated CO$_2$ levels, heat exposure, and poor ventilation negatively affect decision-making accuracy, increase fatigue, and exacerbate respiratory symptoms—effects that are particularly pronounced in children. The **ESC.AI** platform addresses these risks through continuous environmental monitoring supported by edge-based inference mechanisms that evaluate sensor readings against health-aware thresholds and short-term temporal trends to enable timely feedback and intervention.

#### Sensor deployment and system architecture

Each ESC.AI-enabled bus integrates a suite of high-precision sensors, including CO$_2$ sensors based on Non-Dispersive Infrared (NDIR) technology for assessing ventilation quality^39^. Temperature and humidity are monitored using digital thermo-hygrometers to assess thermal comfort conditions. Sensor data are collected via ESP32-based microcontrollers and processed locally using bus-mounted edge-computing units, enabling low-latency analysis without reliance on continuous cloud connectivity^15^.

#### Threshold classification and health-based zones

ESC.AI incorporates real-time environmental monitoring protocols aligned with international standards published by the WHO, ASHRAE, and ISO. Key parameters, such as CO$_2$ concentration, are classified into health-based zones: green (400–800 ppm, indicating adequate ventilation), red (>1000 ppm, associated with increased fatigue and cognitive impairment risk), and blue (<400 ppm, indicating potential over-ventilation). Temperature zones are defined with green representing $20-26^\circ$C and red indicating temperatures above $30^\circ$C, which are linked to elevated risks of heat stress and asthma exacerbation. Relative humidity is maintained within the recommended 40–60% RH range according to CDC guidelines.

Environmental anomalies are detected within a low-latency time frame suitable for real-time intervention, enabling prompt corrective actions when unsafe conditions arise. Threshold-based classification is complemented by short-term temporal analysis to reduce false alarms and ensure alerts correspond to sustained deviations rather than transient fluctuations. These measures support student well-being and learning readiness by mitigating environmental factors that negatively influence memory retention, concentration, and emotional regulation.

#### Sensor selection and deployment rationale

The sensing architecture of the proposed **ESC.AI** framework was designed following a requirements-driven methodology that carefully distinguishes between wearable and embedded sensor modalities. Sensor selection was guided by multiple criteria, including measurement accuracy, robustness in mobile environments, deployment feasibility, user acceptance (particularly in child-centered applications), cost-effectiveness, and compatibility with edge-based artificial intelligence processing.

**Wearable Sensors Rationale** Wearable sensors were selected for physiological measurements that require direct and continuous contact with the human body to ensure reliable signal acquisition. In particular, electrocardiography (ECG) monitoring using modules such as the AD8232 necessitates stable electrode–skin contact to accurately capture cardiac electrical activity and derive clinically relevant indicators such as heart rate variability (HRV). Alternative approaches, including seat-embedded capacitive ECG sensors, were considered; however, these methods are highly sensitive to clothing thickness, body posture, and motion artifacts, which are common in school bus environments. Consequently, wearable ECG sensing was adopted to achieve higher signal fidelity and robustness.

Similarly, peripheral oxygen saturation (SpO$_2$) monitoring relies on optical photoplethysmography, which requires controlled sensor placement and consistent optical coupling. No reliable environmental proxy exists for estimating blood oxygen levels, making direct wearable measurement essential for accurate detection of hypoxia-related conditions. Wearable devices also enable individualized physiological monitoring without interference from surrounding passengers.

For biometric authentication, tissue impedance–based identification mechanisms were selected due to their inherent liveness detection properties and resistance to spoofing attacks. Compared to conventional fixed fingerprint scanners, impedance-based techniques reduce vulnerabilities associated with latent fingerprint replication and improve reliability in high-throughput boarding scenarios involving children.

**Embedded Sensors Rationale** Embedded sensors were employed for parameters that represent shared environmental or vehicle-level conditions rather than individual physiological states. Cabin air quality, measured using non-dispersive infrared (NDIR) CO$_2$ sensors such as the MH-Z19B, reflects a collective environmental property influenced by passenger density and ventilation efficiency. Embedding these sensors at strategic cabin locations enables representative and continuous monitoring without requiring individual devices.

Temperature and humidity sensors (e.g., DHT22) were similarly embedded to capture overall thermal comfort conditions affecting all occupants. Personal temperature sensors were deemed unnecessary, as environmental comfort is primarily governed by cabin-level conditions rather than individual microclimates.

Vision-based behavioral monitoring relies on fixed RGB and infrared cameras installed within the driver and passenger cabins. Embedded camera placement provides a stable field of view, consistent illumination conditions, and wide-area coverage necessary for detecting driver distraction, drowsiness, and student-related safety events. Wearable cameras were considered impractical due to usability constraints, privacy concerns, and potential discomfort, particularly for children.

Global positioning system (GPS) modules, such as the NEO-6M, were deployed at the vehicle level since routing optimization, fleet tracking, and arrival time estimation operate on a bus-level granularity. Individual GPS devices were therefore unnecessary for the intended routing and operational objectives. Alcohol detection sensors (MQ-3) were embedded within the driver cabin to support pre-departure and continuous sobriety assessment, as monitoring a single driver does not justify wearable deployment.

**Deployment Strategy** The combined use of wearable and embedded sensors enables complementary sensing capabilities within the ESC.AI framework. Wearable devices provide individualized physiological monitoring where high-fidelity body contact is required, while embedded sensors capture shared environmental, behavioral, and vehicular states. This hybrid deployment strategy minimizes redundancy, reduces user burden, and enhances system scalability while maintaining comprehensive situational awareness in dynamic school transportation environments.

#### Impact on student health and learning readiness

Suboptimal air quality in school transportation environments has been shown to impair cognitive functions, including memory retention, concentration, and emotional regulation. The ESC.AI platform addresses these risks through real-time detection of poor air-quality events using integrated environmental sensors. When adverse conditions are identified, the system provides corrective recommendations to the driver to restore air quality to acceptable levels. All detected events are logged to enable correlation with behavioral patterns and to support long-term health and safety analytics. This responsive approach is designed to support safer transportation conditions and promote student readiness for learning.

#### Real-time feedback loop

When unsafe environmental conditions are detected, ESC.AI issues audio and visual alerts to the driver to ensure immediate awareness of potential hazards^40^. The system presents targeted mitigation recommendations, such as adjustments to ventilation rates, to restore desired environmental parameters. Event data are logged and synchronized with cloud-based systems to support trend analysis, anomaly frequency assessment, and long-term performance evaluation^39^. This integrated workflow enables both immediate corrective intervention and continuous refinement of safety protocols within the school transportation environment.

#### Predictive environmental analytics

ESC.AI’s ongoing collection of long-term environmental data lets the system find problems that happen again and again on certain transportation routes, guess when HVAC systems might break down, and help create flexible, seasonal transportation policies. This integrated approach not only supports immediate operational interventions but also enhances the long-term reliability, safety, and responsiveness of school transportation management through data-driven policy optimization and predictive maintenance capabilities. The ESC.AI environmental intelligence subsystem transforms school buses into adaptive, health-responsive microenvironments. By combining international health standards with local edge-based inference, the system provides robust protection against hidden environmental stressors.

### GPS-based monitoring and routing optimization

#### Location and route optimization data

**Real-Time GPS Tracking and Geospatial Integration:** ESC.AI integrates high-frequency GPS telemetry to track school bus locations throughout their routes. Positioning data are processed using GIS-based visualization tools to present real-time bus trajectories, traffic congestion zones, stop density distributions, and temporal travel patterns. Heat mapping and clustering techniques are applied to identify recurrent high-delay and high-risk areas within the transportation network. These insights support informed rerouting decisions aimed at improving travel efficiency and predictability in school transportation operations^6,7^.

#### Safety and security-driven route optimization

A key capability of ESC.AI is its safety-aware routing engine, which extends conventional routing approaches by incorporating risk-based metrics alongside traffic data. The system evaluates neighborhood-level crime statistics, road surface conditions, structural hazards, and environmental risk indicators such as flood-prone areas to inform routing decisions. Routes traversing higher-risk regions may be deprioritized, while bus stop locations are periodically reassessed based on geospatial safety indicators. This adaptive routing strategy is designed to enhance student safety during boarding, transit, and drop-off activities across dynamically changing transportation networks^18^.

#### Data flow and cybersecurity in bus tracking

Data acquisition in ESC.AI begins at the Perception Layer, where heterogeneous streams—including environmental, physiological, IoT, and GPS data—are aggregated. Prior to transmission across system layers, incoming data undergo validation, standardization, and noise reduction to improve reliability and coherence. Data flows are protected using layered cybersecurity mechanisms, including AES-based encryption, PKI-enabled device authentication, and lightweight SIMECK cipher protocols. These measures are intended to preserve data integrity and confidentiality while reducing exposure to unauthorized access, providing a secure foundation for downstream analytics and operational decision-making^41^.

### Routing optimization algorithm

#### Ant Colony Optimization (ACO) for school bus routing

ESC.AI employs Ant Colony Optimization (ACO), a bio-inspired metaheuristic algorithm, to address the school bus routing problem under multiple and potentially conflicting objectives. The routing formulation considers travel time, total route length, fuel efficiency^24^, and exposure to unsafe areas as optimization criteria. In the proposed framework, artificial pheromone trails are updated iteratively based on route quality, while heuristic information incorporates traffic conditions and safety-related constraints. This enables the algorithm to adapt route selection in response to evolving traffic patterns and contextual risk indicators, supporting flexible and resilient school transportation planning^42^.

**Dataset Description:** The ACO-based routing engine was evaluated using a synthesized geospatial dataset representing 250 student nodes and 15 school destinations distributed across the Alexandria metropolitan area. Each node includes GPS coordinates, road network connectivity, and traffic density attributes derived from historical data sources. This dataset enables realistic modeling of urban routing constraints while supporting safety-aware optimization scenarios.

#### Geospatial analysis of bus routes

**Fig. 7:**
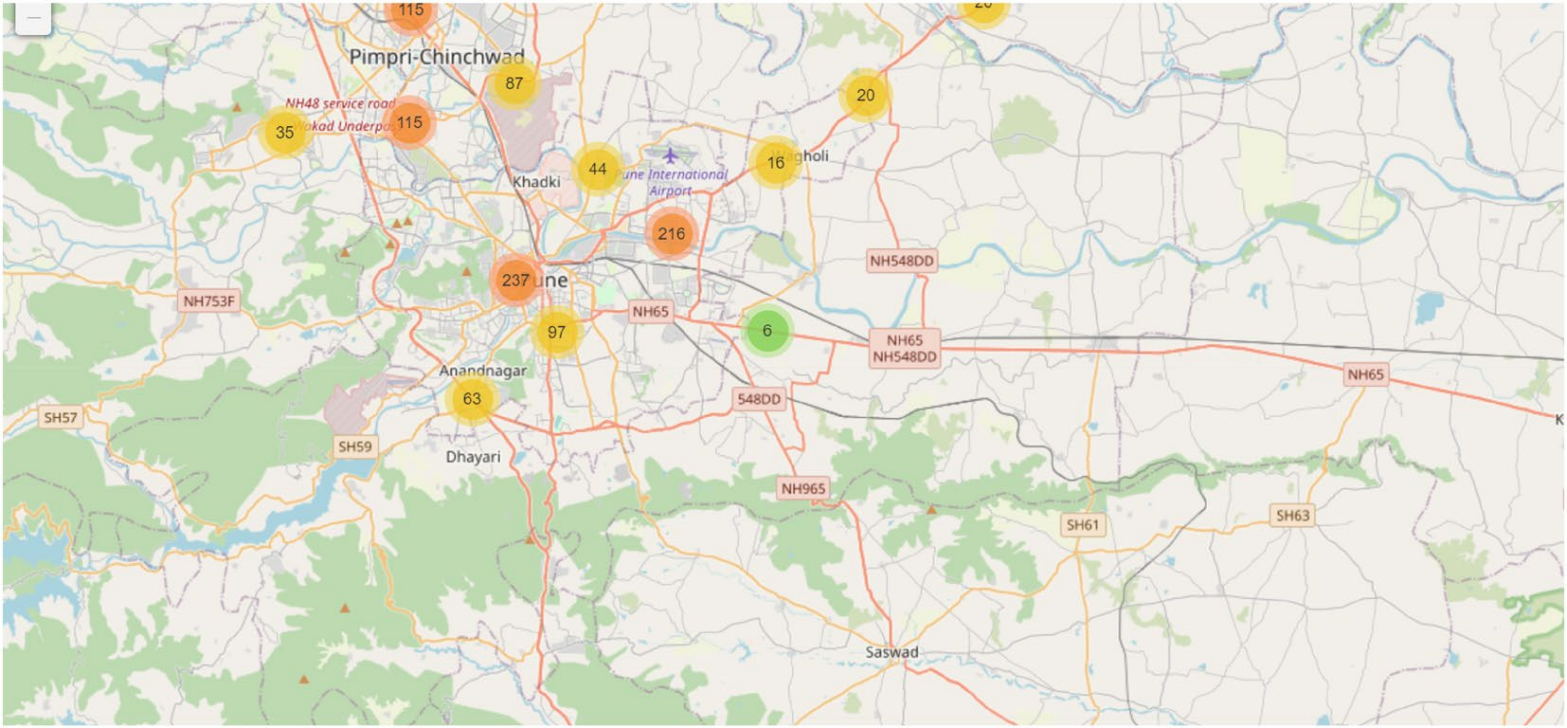
Geospatial analysis of bus routes with marked bus stops and traffic zones. The map shows various bus stops and their respective traffic zones. The color coding indicates the density of bus stops in various city areas. The number on each circle represents the total count of bus stops in that area.

Geospatial analysis of school bus routes provides insight into bus stop distribution, traffic density, and congestion-prone regions, which are critical for effective route optimization. By leveraging real-time GPS tracking, the system identifies areas associated with recurrent delays and supports adaptive routing adjustments based on traffic flow, road conditions, and stop density^42^. Figure 7 illustrates the spatial distribution of bus stops and corresponding traffic zones. Color coding indicates stop density, with darker red regions representing higher concentrations and lighter shades indicating lower density. The numeric labels denote the number of stops within each zone, supporting informed decisions regarding route frequency and service coverage.

**Fig. 8:**
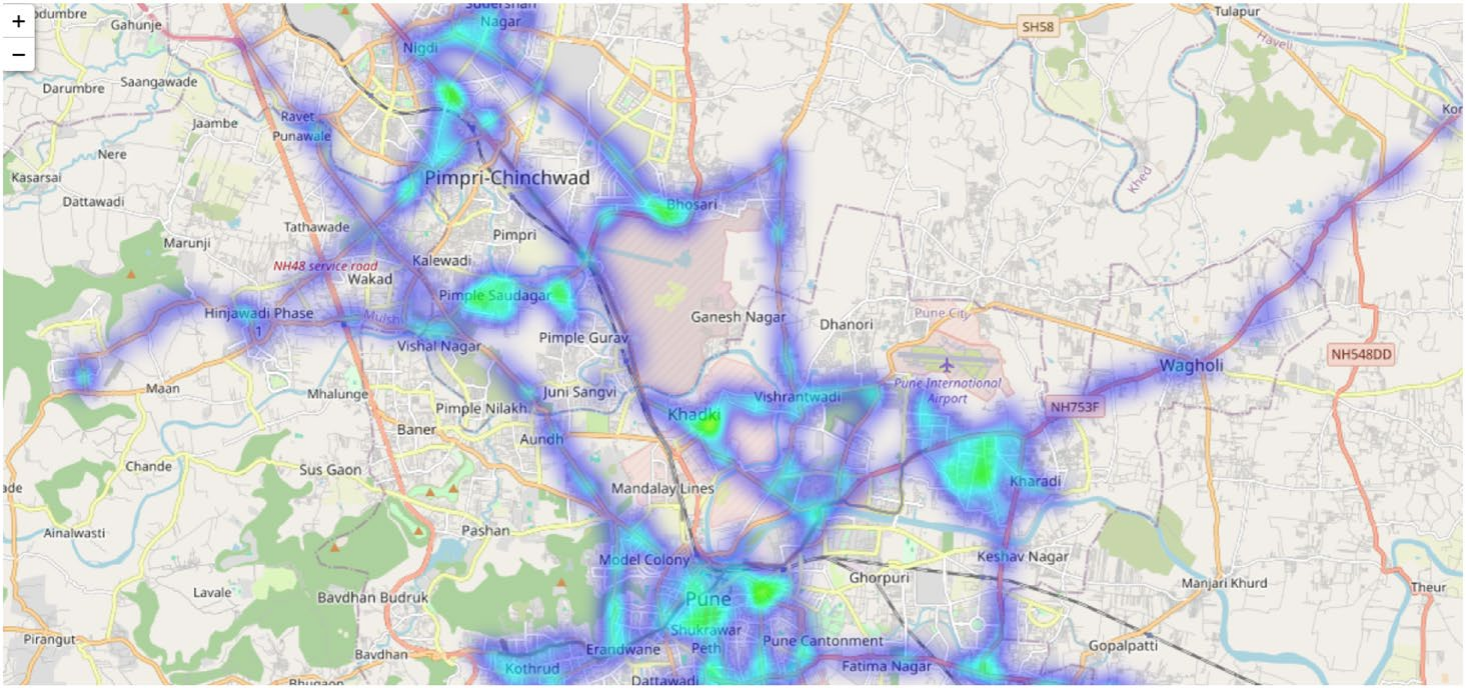
Heatmap representation of traffic density around bus routes. The darker blue regions indicate areas with low traffic, while the bright green and yellow regions represent high-traffic zones. This visualization is useful for identifying areas prone to delays and optimizing bus routes to avoid congested zones.

Figure 8 presents a traffic density heatmap for bus routes using a color gradient to represent varying traffic volumes. Darker blue regions correspond to low-traffic areas, while green and yellow regions indicate higher traffic density. This visualization highlights congestion-prone segments that may affect route efficiency. Integration of real-time GPS and traffic data allows the routing module to adapt paths in response to current traffic conditions, supporting more informed routing decisions^42^.

**Fig. 9:**
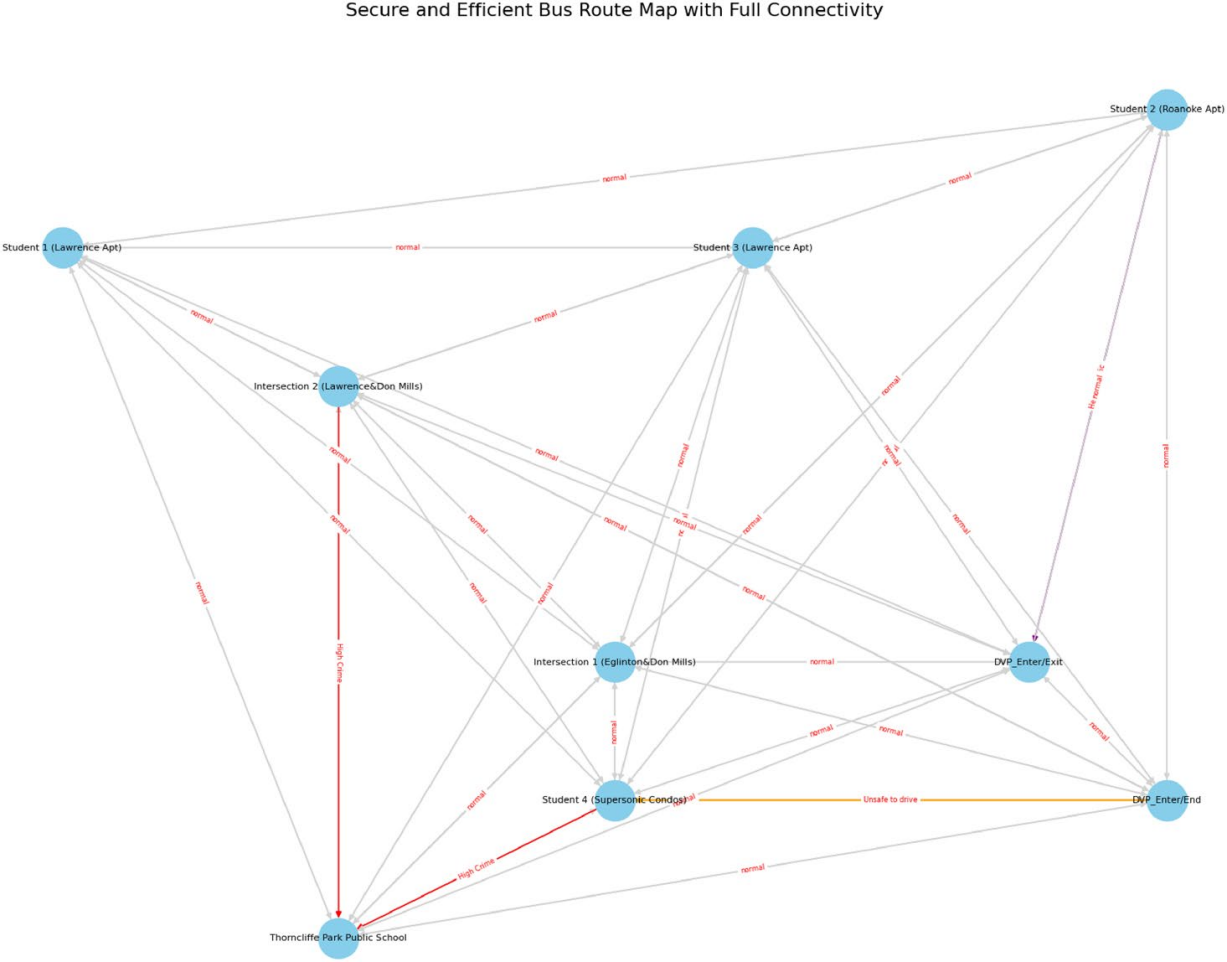
Graph of optimized bus routes with traffic constraints and safety considerations. Red lines indicate the optimal path, while other edges represent alternative routes that were evaluated but not chosen due to traffic conditions, high crime zones, or unsafe roads.

Optimized routing under traffic and safety constraints is illustrated in Fig. 9, where the transportation network is modeled as a graph. Nodes represent student residences, intersections, and school locations, while edges correspond to feasible road segments. Red edges indicate routes selected by the ACO algorithm based on combined traffic and safety heuristics^9^. Alternative routes evaluated but not selected are shown in black. Safety constraints include traffic congestion near major intersections high-crime regions^11^, and unsafe road segments. This formulation enables route selection that balances efficiency considerations with safety awareness.

The ACO-based routing framework integrates geographic information, real-time traffic inputs, and GPS monitoring to support adaptive route planning under dynamic conditions. Cybersecurity mechanisms, including edge-based processing and secure data handling, are incorporated to protect sensitive routing and location data and to enhance system resilience against cyber threats^43^.

**Table 2:** Bus Stop Nodes with Coordinates.

Node	Latitude	Longitude
Home1	3.1456	2.6789
Home2	24.8765	−14.9876
MainRoad1	49.7543	11.1023
Intersection1	−19.6789	41.2301
Hospital1	6.1234	5.8456
School	−34.7890	85.4321

Continuous monitoring of safety-related data enables the routing module to account for hazardous areas during route computation. The node-based graph representation, summarized in Table 2, defines key geographic locations and their coordinates, supporting consistent geospatial computation and route evaluation^9,25,42^.

###  Tissue impedance sensing for biometric identity verification

**Fig. 10:**
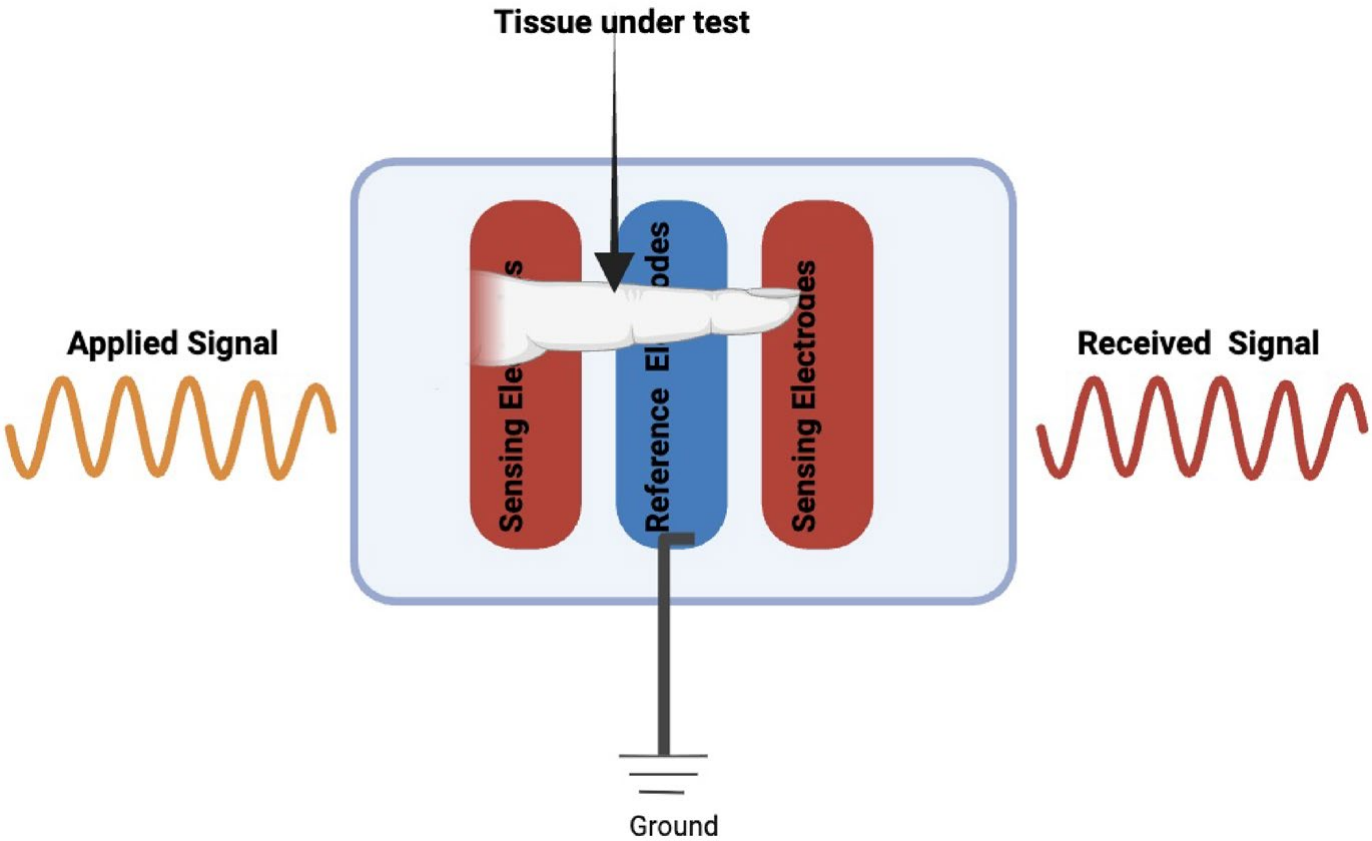
Impedance measurement setup showing tissue under test placed above sensing and reference electrodes, where an applied signal is transmitted through the tissue and detected as a received signal for analysis. The ground connection ensures an accurate reference potential.

**Fig. 11:**
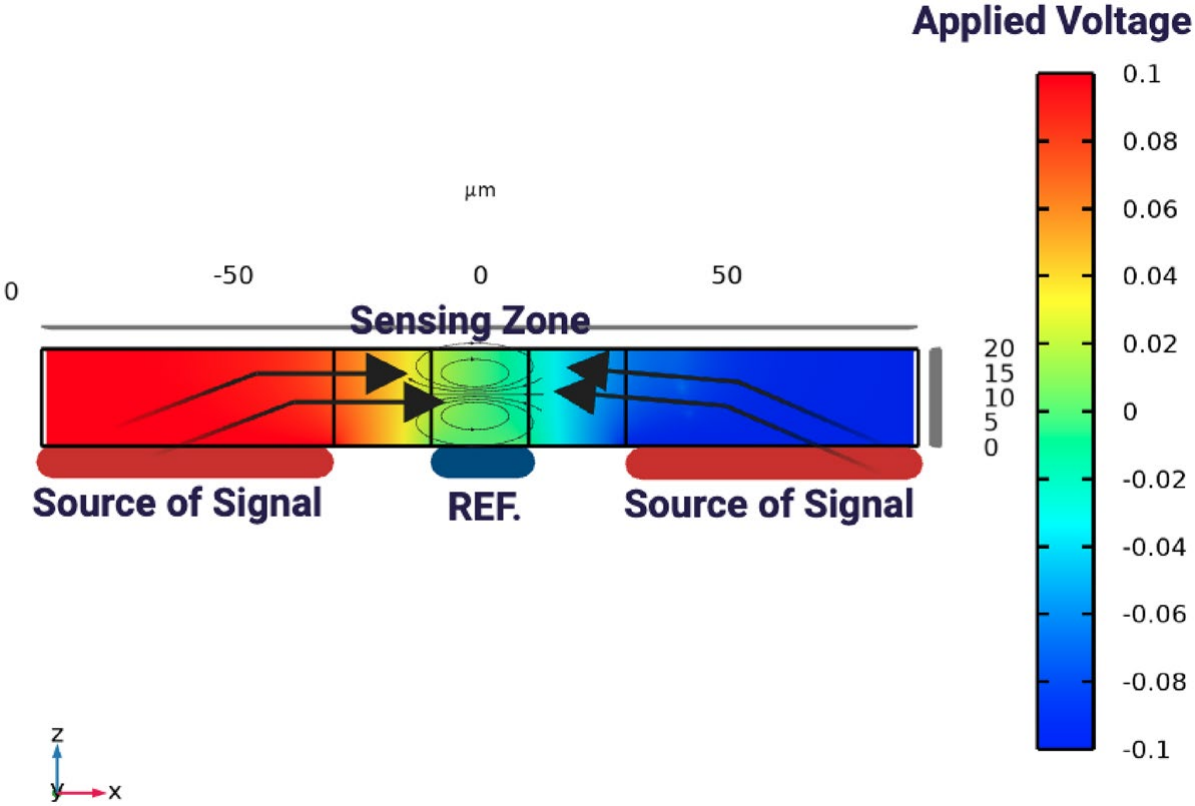
Schematic representation of electric field lines between sensing and reference electrodes, illustrating how the applied field interacts with the finger above the electrodes. The spatial arrangement of the field lines governs how the system probes the dielectric properties of the tissue, enabling characterization of its permittivity and conductivity. This setup is designed to sensitively define and quantify the dielectric features of biological samples under test.

In the proposed system, interdigitated microelectrodes (IDEs) are used, as shown in Figs. 10 and 11. The finger is positioned so that it fully covers the primary excitation and sensing electrodes, ensuring stable coupling to the tissue. The electrodes are driven using the Analog Discovery module, which provides both the excitation signal and the readout of the impedance fluctuations. A safe voltage level (0.1 Vp) and a frequency sweep within the range (50 Hz to 5 MHz) are applied to extract the characteristic impedance features of different tissue types.

Tissue impedance sensing exploits the fact that biological tissues exhibit frequency-dependent electrical properties governed by cellular structure, water content, and ionic composition. Variations in epidermal thickness, sweat gland density, vascularization, and subcutaneous tissue distribution introduce subject-specific impedance signatures. When an alternating electric field is applied across the IDE array, current pathways are modulated by tissue permittivity and conductivity, resulting in distinct impedance spectra that are difficult to replicate artificially.

The selected excitation frequency range (50 Hz–5 MHz) enables characterization of multiple dispersion regions, including electrode polarization effects at low frequencies and membrane capacitance behavior at mid-to-high frequencies. This broad spectral coverage allows extraction of discriminative features associated with both extracellular and intracellular electrical properties. Figure 14 illustrates the variation in terminal current as the finger moves across the electrode array. The lowest current is observed when the finger is positioned directly above the sensing and reference electrodes. This behavior is consistent with the impedance-based sensing principle, where the presence of biological tissue increases impedance and consequently reduces current flow. These results confirm the sensitivity of the system to biological interface effects and validate the effectiveness of the electrode-based detection mechanism.

**Fig. 12:**
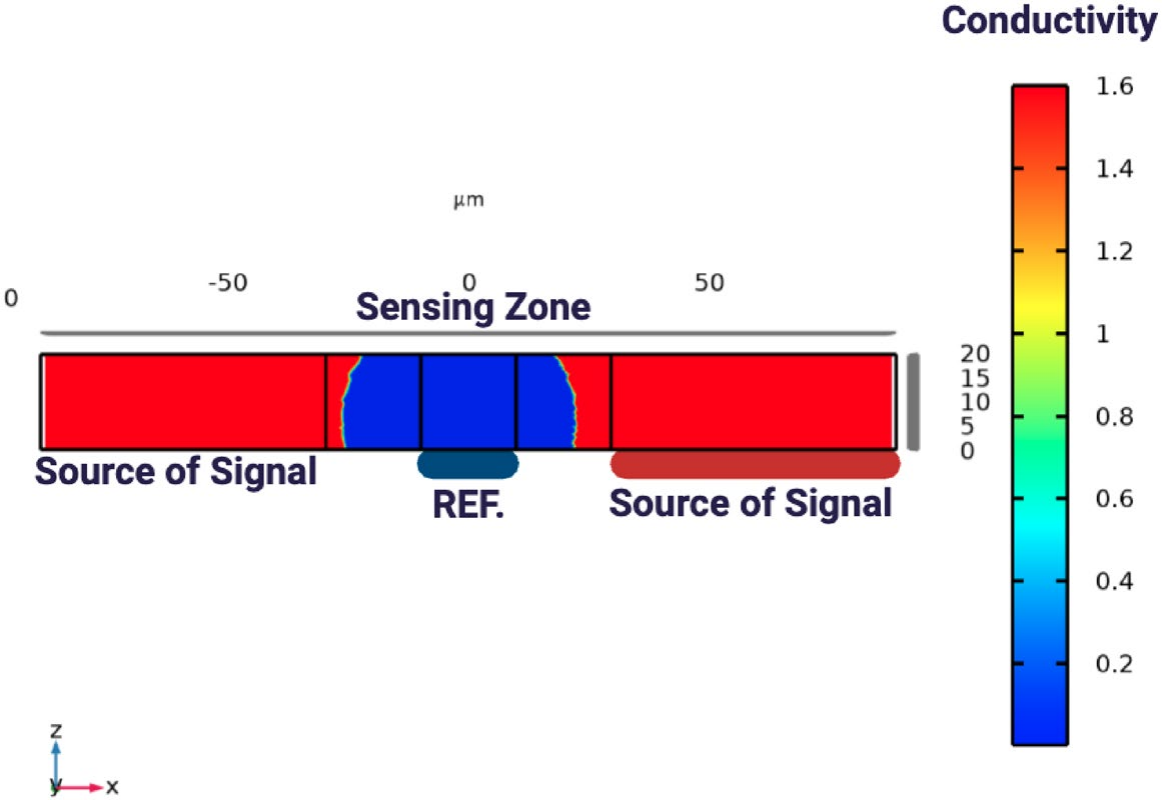
IDE sensor schematic illustrating current variation due to finger placement above the electrodes, where biological tissue reduces local conductivity relative to air or buffer medium. The finger disrupts the signal pathway, increases impedance, and lowers terminal current, confirming the system’s ability to detect spatial changes in conductivity at the biological–electrode interface.

**Fig. 13:**
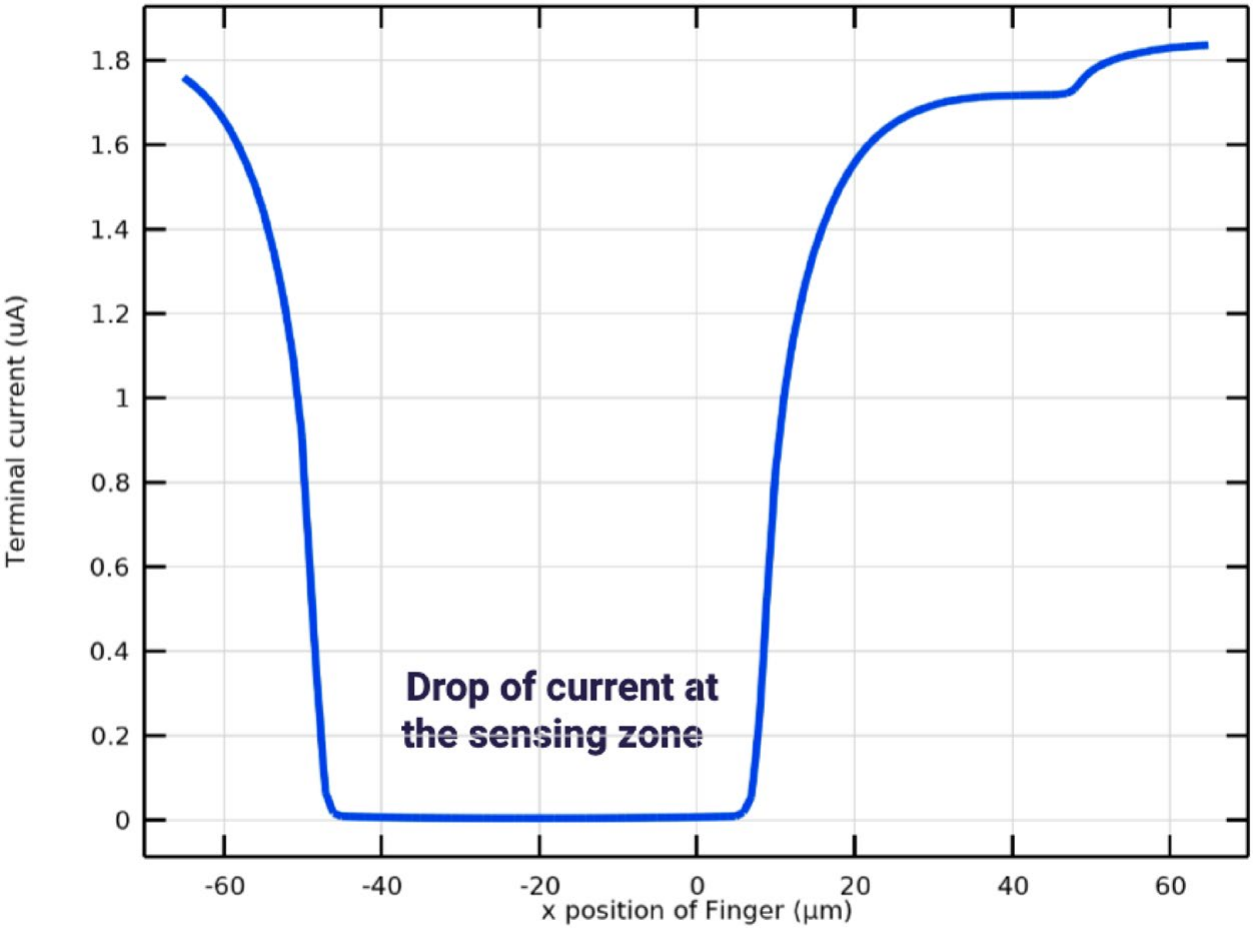
Terminal current versus finger position response showing current reduction when the finger is placed above the sensing electrodes, indicating increased impedance due to the presence of tissue. The lowest current occurs at the central electrode region, where the finger blocks signal flow, demonstrating sensitivity of the measurement system to biological material at electrode interface.

The electrical behavior of the sensing system is illustrated in Fig. 13, which models current flow and impedance variation under different finger positions. Figure 12 further depict conductivity perturbations and electric-field redistribution caused by tissue presence. These simulations validate the sensitivity of the IDE configuration prior to experimental measurements and confirm that finger placement within the sensing zone produces measurable impedance deviations.

**Fig. 14:**
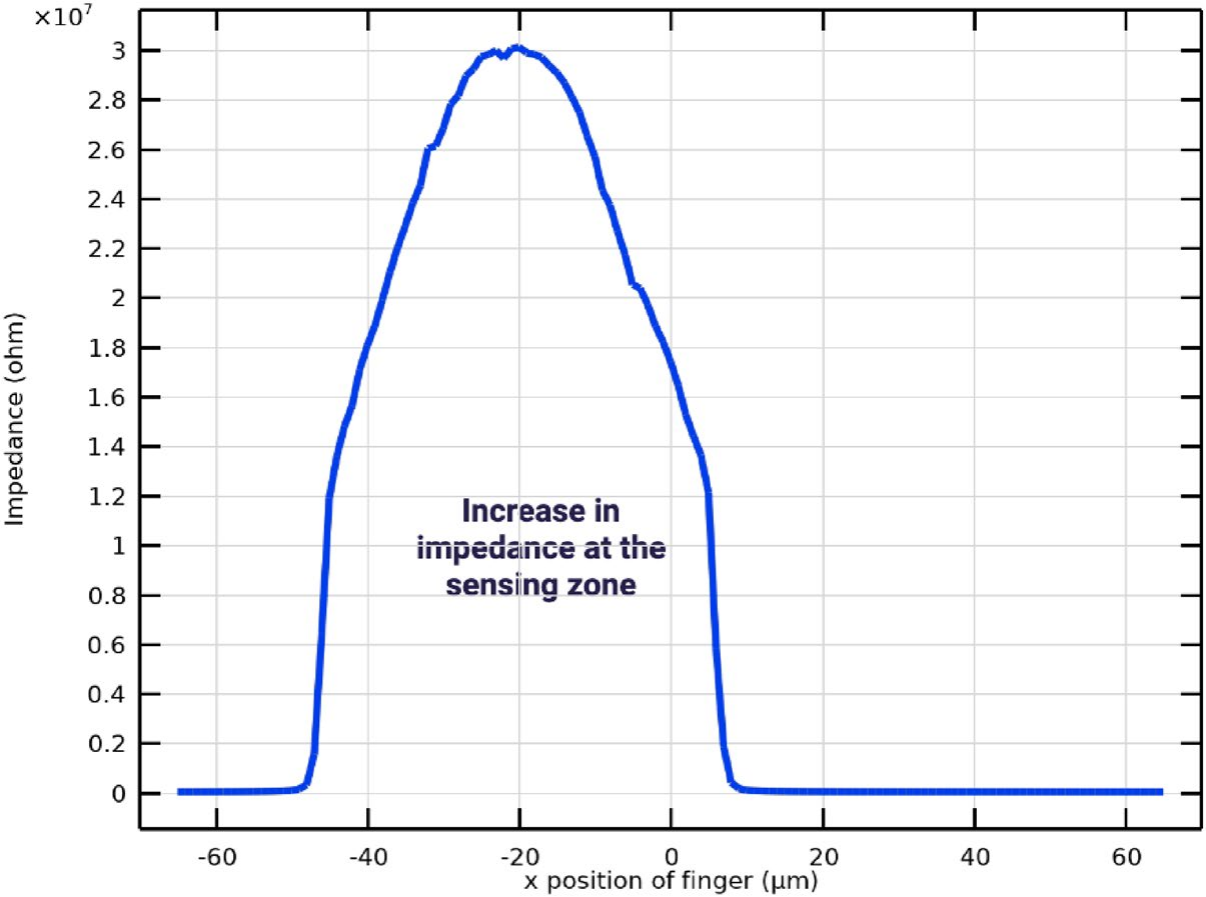
Terminal current variation as the finger moves across the electrode array, with the lowest current observed when the finger is directly above the sensing and reference electrodes. This aligns with the impedance-based measurement concept, where tissue presence at the electrodes blocks signal flow and increases impedance, resulting in reduced current and confirming the sensitive detection of biological interface effects.

Impedance measurements were acquired from five fingers across multiple recording sessions, with each acquisition generating complex impedance values across the excitation frequency sweep. From each trial, ten ratiometric impedance features were computed by normalizing inter-electrode responses, reducing sensitivity to absolute contact pressure and environmental variation. These feature vectors were used to train Random Forest and XGBoost classifiers under a cross-validation framework, achieving classification accuracies of 92% and 91%, respectively.

**Data Acquisition Protocol and Spectral Characteristics**

Impedance data were collected from five distinct subjects across multiple acquisition sessions in order to capture both intra-subject repeatability and inter-subject variability. Each recording session consisted of repeated impedance sweeps while the subject placed a finger naturally on the interdigitated electrode array, without applying excessive force. This protocol was intentionally designed to reflect realistic usage conditions in mobile and semi-uncontrolled environments such as school buses, where precise pressure control cannot be enforced.

For each subject, impedance spectra were recorded over the full excitation range from 50 Hz to 5 MHz. The magnitude of the complex impedance, |*Z*(*f*)|, was computed at each frequency point and subsequently averaged across repeated trials. This averaging process mitigated transient fluctuations arising from micro-movements, contact impedance variations, and short-term physiological changes, thereby improving measurement stability and reproducibility.

**Fig. 15:**
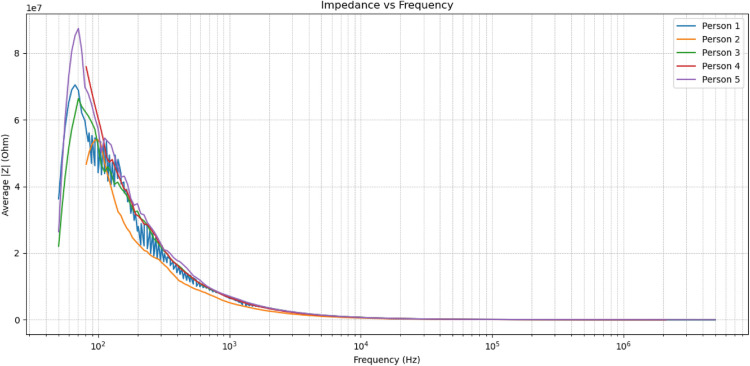
Averaged impedance magnitude as a function of frequency for all subjects.

Figure 15 presents the averaged impedance magnitude as a function of frequency for all five subjects. At low frequencies ($<200$ Hz), all impedance curves exhibit elevated magnitudes, which are primarily governed by electrode polarization effects and the highly resistive nature of the outer skin layers, particularly the stratum corneum. In this regime, current flow is constrained to superficial pathways, resulting in high impedance values with limited discriminative utility.

As frequency increases, a pronounced monotonic decay in impedance magnitude is observed across all subjects. This behavior corresponds to increased capacitive coupling across cell membranes and reduced reactance at the electrode–tissue interface, allowing current to penetrate deeper into conductive tissue layers. Within this transition region, subject-specific differences become increasingly apparent.

Notably, the mid-frequency range exhibits distinct and repeatable subject-specific impedance profiles, characterized by variations in peak magnitude, decay slope, and spectral curvature. These differences arise from intrinsic physiological factors, including epidermal thickness, tissue hydration, sweat gland density, vascularization, and subcutaneous composition. Importantly, these characteristics are largely invariant to superficial surface features, reinforcing their suitability for biometric identification.

At higher frequencies ($>100$ kHz), the impedance curves begin to converge across subjects as capacitive reactance diminishes and bulk tissue conductivity dominates the electrical response. In this regime, inter-subject variability is reduced, resulting in lower discriminative power for biometric classification. This convergence behavior is consistent with established bioimpedance models and further validates the physical plausibility of the measured spectra.

The observed spectral behavior directly informed the feature extraction strategy employed in this work. Feature importance analysis conducted using both Random Forest and XGBoost classifiers consistently identified the 8–11 kHz frequency band as the most discriminative region for inter-subject classification. This frequency range represents a balance point where resistive and capacitive tissue properties jointly influence signal propagation, maximizing sensitivity to physiological variability while maintaining robustness against contact inconsistencies.

To enhance reliability under real-world operating conditions, ratiometric impedance features were employed alongside frequency-domain averaging. This normalization strategy effectively suppresses global scaling effects caused by finger pressure, electrode contact area, and environmental drift, while preserving relative spectral differences between fingers and subjects. Experimental evaluation confirmed stable classification performance under moderate variations in finger placement and contact force.

By integrating tissue-level impedance biosensing with machine learning-based classification, this module introduces a physiological authentication layer that extends beyond conventional biometrics such as RFID cards or optical fingerprint systems. Unlike optical methods, which are sensitive to illumination, surface contamination, and spoofing artifacts, impedance signatures are derived from intrinsic volumetric tissue properties, providing inherent liveness detection and increased resistance to replay attacks. Furthermore, the absence of stored biometric images supports privacy-preserving identity verification, making the proposed approach particularly suitable for secure and ethical deployment in intelligent school transportation systems.

**Biometric Classification Performance**

To provide a more comprehensive assessment of biometric authentication performance, standard classification metrics were evaluated under a stratified cross-validation protocol. In addition to overall accuracy, precision, recall, and F1-score were computed to quantify class separability and authentication reliability.

The Random Forest classifier achieved a precision of 93%, recall of 91%, and an F1-score of 92%, while the XGBoost model achieved a precision of 92%, recall of 90%, and an F1-score of 91%. The balanced performance across these metrics indicates consistent discrimination between enrolled subjects without bias toward any single class, as summarized in Table 3.

False acceptance and false rejection rates remained low across validation folds, suggesting stable authentication behavior under moderate variations in finger placement and contact conditions. These results confirm that the reported accuracy reflects robust biometric separability rather than dataset bias.

**Table 3:** Biometric authentication performance metrics for tissue impedance–based identification.

Classifier	Accuracy	Precision	Recall	F1-score
Random Forest	92%	93%	91%	92%
XGBoost	91%	92%	90%	91%

**Comparison with Optical Biometric Systems** Conventional biometric authentication systems deployed in transportation and access-control settings predominantly rely on optical modalities, including fingerprint imaging, facial recognition, or iris scanning. While these approaches have demonstrated high accuracy under controlled conditions, their performance is often sensitive to illumination variability, surface contamination, camera occlusion, and user cooperation. In mobile environments such as school buses, these constraints are exacerbated by motion, vibration, variable lighting, and hygiene considerations.

In contrast, the proposed tissue-impedance–based biometric approach operates independently of optical conditions and does not require image acquisition. Identity features are derived from intrinsic electrical properties of biological tissue, which are influenced by internal physiological structure rather than surface patterns. This characteristic provides increased robustness to environmental lighting changes, finger surface contamination, and minor positioning variations. Moreover, because no visual biometric images are captured or stored, the approach inherently supports privacy-preserving authentication and reduces the risk of biometric data misuse or replay attacks commonly associated with image-based systems. A comparative summary of optical and tissue-impedance–based biometric systems is provided in Table 4.

**Table 4:** Comparison between optical biometric systems and tissue-impedance–based authentication.

Criterion	Optical biometrics	Tissue impedance biometrics
Lighting dependency	High	None
Surface contamination sensitivity	High	Low
Privacy risk (image storage)	High	Low
Spoofing susceptibility	Moderate	Low
Environmental robustness	Moderate	High
Mobile deployment suitability	Limited	High

Compared to traditional optical biometric systems, the proposed impedance-based authentication provides a privacy-preserving and environmentally robust alternative that is particularly suited to mobile and resource-constrained transportation environments.

## Results

### Driver Monitoring System (DMS)

The Driver Monitoring System was built using a YOLOv8 model trained specifically for in-cabin driver behavior analysis. The system detects five critical driving behaviors: *Open Eye*, *Closed Eye*, *Cigarette*, *Phone*, and *Seatbelt*. These behaviors represent high-risk indicators associated with driver fatigue, distraction, and safety non-compliance in school transportation environments.

#### Model configuration

The following settings were used to configure YOLOv8 for real-time edge inference:

- **Batch Size:** 16 (optimized for GPU memory constraints)
- **Learning Rate:** Initialized at 0.01 and decayed using a cosine schedule
- **Anchor Boxes:** Customized to match the aspect ratios of eye regions, handheld objects, and seatbelt geometries
- **Export Format:** ONNX-compatible weights for deployment within the ESC.AI edge inference framework

#### Training and evaluation

The model was trained for 100 epochs using the Adam optimizer. Data augmentation techniques were applied to enhance robustness against variations in illumination, pose, and partial occlusion. Performance evaluation employed standard object detection metrics:

- **Precision:** 89.4%
- **Recall:** 87.2%
- **mAP@0.5:** 92.1%
- **mAP@[0.5:0.95]:** 67.6%

The model demonstrated strong detection performance for mobile phone usage and seatbelt compliance. Reduced performance at higher IoU thresholds was primarily observed for eye-state classification under low-light or partially occluded conditions, particularly when distinguishing between open and closed eyes. Class-wise analysis indicated that phone detection achieved the highest accuracy, while confusion was most prominent between eye-state classes in darker environments.

#### Comparison with prior YOLO-based driver monitoring systems

To contextualize the performance of the proposed YOLOv8-based Driver Monitoring System (DMS), a comparison was conducted against representative YOLO-based approaches reported in the literature. In particular, Wang et al^44^. proposed an improved YOLOv5 architecture for driver attention detection, achieving a mean Average Precision (mAP) of 86.3% while maintaining real-time performance at 55 FPS with a compact model size of 17.5 MB.

Compared to baseline YOLOv4, YOLOv5, and YOLOv7 implementations, the improved YOLOv5 model demonstrated superior detection accuracy, with gains of up to 2.4% mAP over the original YOLOv5, while remaining significantly lighter than YOLOv4 and YOLOv7. Class-wise evaluation further showed notable improvements in detecting distraction-related behaviors such as smoking (+4%) and mobile phone usage (+3%), highlighting the effectiveness of architectural refinements for fine-grained in-cabin behavior analysis.

The proposed ESC.AI DMS builds upon these findings by leveraging the more recent YOLOv8 architecture, which further improves detection robustness and inference efficiency under low-light and partially occluded in-cabin conditions. While Wang et al^44^. focused primarily on driver attention behaviors, ESC.AI extends the detection scope to include seatbelt compliance and eye-state monitoring within a unified, edge-deployable framework tailored for school transportation safety.

#### CNN architecture details

The YOLOv8 architecture employs a convolutional neural network (CNN) backbone optimized for real-time vision tasks. Residual connections and a Cross Stage Partial (CSP) design are used to improve gradient flow while reducing computational overhead. Multi-scale feature fusion is achieved through a Path Aggregation Network (PANet), enabling effective detection of small or partially occluded objects such as eyelids and handheld devices.

The detection head utilizes an anchor-free prediction strategy to jointly estimate bounding box coordinates and class probabilities. By directly regressing object center points, width, and height, the model achieves a balance between inference speed and localization accuracy, making it well suited for dynamic in-cabin conditions with variable illumination and occlusion.

**Fig. 16:**
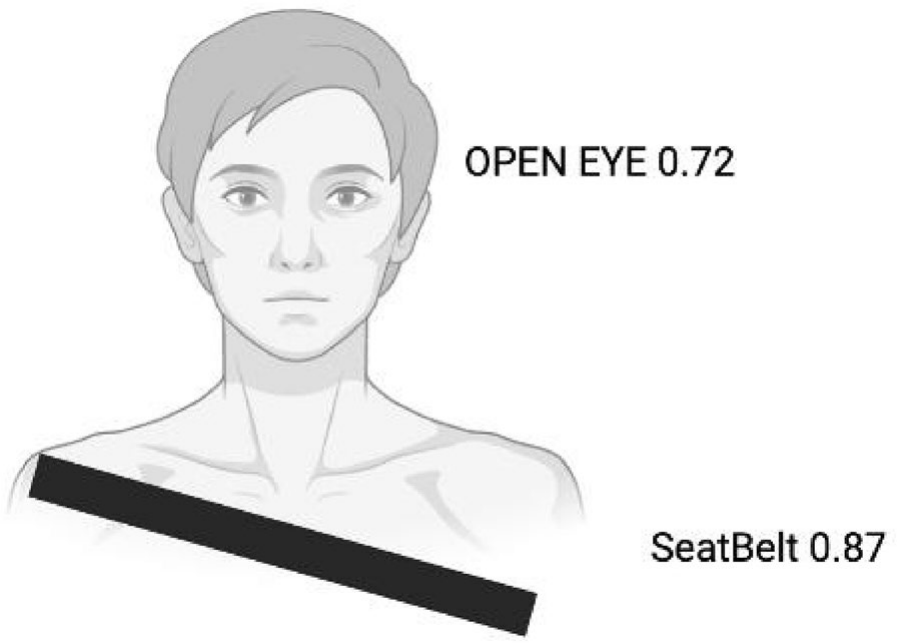
YOLOv8 inference output of the Driver Monitoring System showing detection of eye state and seatbelt usage with associated confidence scores under typical cabin illumination conditions.

### Monitoring the environment: CO$_2$ and temperature

The **ESC.AI** platform incorporates a real-time environmental monitoring subsystem designed to detect air quality deviations and temperature anomalies within the bus cabin during student transportation. Environmental sensing was conducted over a continuous evaluation period using MH-Z19B CO$_2$ sensors and DHT22 temperature modules deployed near student seating areas to capture representative in-cabin conditions.

The subsystem combines standards-based thresholding with a lightweight machine learning classifier to support timely safety notifications. CO$_2$ concentrations were continuously monitored and categorized into ventilation zones aligned with ASHRAE and WHO guidelines^45,46^: Green (350–1000 ppm), indicating adequate ventilation; Red Alert (>1100 ppm), associated with increased fatigue, reduced cognitive performance, and discomfort; and Blue Alert (<350 ppm), indicating potential over-ventilation, which may cause thermal discomfort or respiratory irritation.

Temperature measurements were evaluated using pediatric thermal comfort criteria, with Green ($\le$26$^\circ$C) indicating acceptable cabin conditions and Red Alert (>26$^\circ$C) corresponding to elevated risk of heat stress, dehydration, or agitation^47^. The classification performance of the environmental risk detection subsystem is summarized in Table 5.

**Table 5:** Classification report for identifying environmental risks using temperature and CO$_2$ sensor data.

Class	Precision	Recall	F1-score	Support
High Risk	0.77	0.65	0.71	532
Normal Risk	0.95	0.97	0.96	3580
Accuracy	0.93			
Macro Avg	0.86	0.81	0.83	4112
Weighted Avg	0.93	0.93	0.93	4112

#### Comparison with prior in-vehicle environmental monitoring systems

To contextualize the performance of the ESC.AI environmental monitoring subsystem, a comparison is drawn with prior machine learning–based in-vehicle air quality monitoring systems. Goh et al^48^. proposed a real-time in-vehicle air quality monitoring framework using machine learning prediction algorithms, achieving high classification accuracy (approximately 95–97%) and demonstrating strong generalization across different driving and behavioral conditions.

Their system showed improved performance compared to traditional CNN-based and baseline models, while maintaining real-time operational capability. The study confirmed that deep learning–based models can reliably analyze in-cabin environmental conditions and support intelligent transportation systems through accurate prediction of air quality and safety-related risks.

In comparison, the ESC.AI platform extends this paradigm by integrating both standards-based thresholding and lightweight machine learning classification for CO$_2$ and temperature monitoring. This hybrid strategy improves robustness under sensor noise and varying ventilation conditions, while enabling edge-deployable, low-latency operation tailored for safety-critical school transportation environments.

### Analyzing breathing sounds for kids

To facilitate the early identification of juvenile respiratory anomalies during school transportation, **ESC.AI** incorporates a respiratory sound analytical subsystem that leverages hybrid deep learning architectures. The subsystem captures breathing signals using a seatbelt-integrated piezoelectric microphone (CM-01B) positioned near the chest for non-contact monitoring. Dedicated signal processing pipelines were employed to suppress cabin noise and vibration, ensuring reliable acquisition under real-world vehicle motion.

Respiratory sound signals were segmented at the breathing-cycle level and processed using Mel-Frequency Cepstral Coefficients (MFCCs) to capture discriminative spectral characteristics. Moderate augmentation strategies, including pitch shifting and time stretching, were applied during training to enhance robustness while preserving physiological relevance.

**Model Design and Feature Extraction:** The Discrete Cosine Transform (DCT) was used to compute MFCCs from the logarithmic power spectrum of each segment:

1 $$\begin{aligned} C_n = \sum _{m=1}^{M} S_m \cos \left[ \frac{n(m - 0.5)\pi }{M}\right] \end{aligned}$$

where $C_n$ denotes the $n^{th}$ MFCC coefficient, $S_m$ represents the log power spectrum, and *M* is the number of frequency bins.

Two hybrid deep learning architectures—CNN–GRU and LSTM–CNN—were evaluated to model both spectral and temporal characteristics of respiratory cycles. The convolutional output is defined as:

2 $$\begin{aligned} O_{i,j} = f\left( \sum _{m,n} I_{i+m, j+n} K_{m,n} + b\right) \end{aligned}$$

where *I* is the input feature map, *K* is the convolution kernel, *b* is the bias term, and $f(\cdot )$ denotes the activation function. Temporal dependencies across breathing cycles were subsequently captured using recurrent units.

**Fig. 17:**
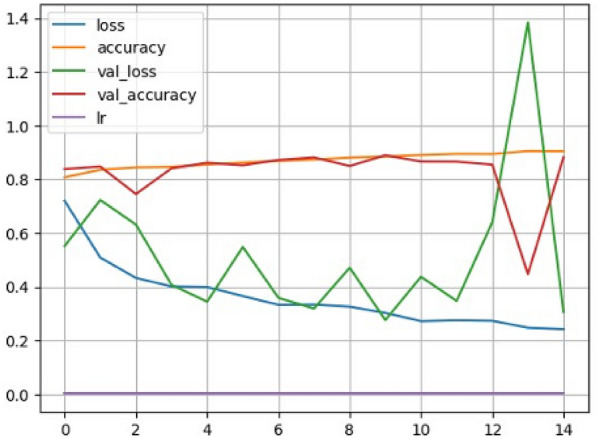
Training and validation accuracy and loss curves for CNN–GRU and LSTM–CNN respiratory sound classification models, showing stable convergence and strong generalization.

Both models were trained for 50 epochs using the Adam optimizer and cross-entropy loss with an 80/20 train–validation split. The LSTM–CNN achieved a validation accuracy of 88.7% and an F1-score of 88.7%, slightly outperforming the CNN–GRU model, which achieved an accuracy of 87.3% and an F1-score of 87.2%. Training and validation curves (Fig. 17) demonstrate stable convergence with minimal overfitting.

**Fig. 18:**
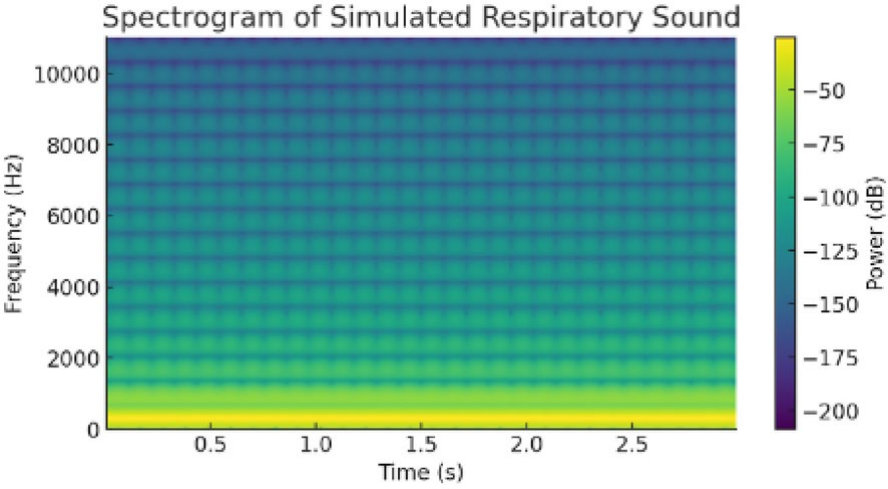
Mel-spectrograms of (**a**) normal and (**b**) abnormal respiratory sounds, highlighting spectral differences between wheeze and crackle events.

As illustrated in Fig. 18, Mel-spectrograms of normal and abnormal respiratory sounds exhibit distinct spectral patterns. Wheezing events show prolonged high-frequency components, whereas crackling episodes appear as short, transient bursts concentrated at lower frequencies.

This subsystem enhances the health intelligence layer of **ESC.AI** by enabling early detection of respiratory abnormalities such as asthma-related airway obstruction and irregular breathing patterns during school bus operation.

### Seizure detection based on EMG

The **ESC.AI** system incorporates a seizure detection module based on electromyography (EMG) to enable rapid and non-invasive screening of convulsive events in pediatric passengers during transportation^49,50^. The module analyzes muscle-activity signals associated with seizure-related motor patterns and applies signal processing and machine learning techniques to distinguish seizure and non-seizure events under real-world conditions.

A 4th-order Butterworth bandpass filter (20–120 Hz) was applied to isolate seizure-relevant muscle components while suppressing low-frequency drift and high-frequency noise. The Root Mean Square (RMS) energy, used as a primary activation descriptor, is defined as:

3 $$\begin{aligned} E_{\text {RMS}} = \sqrt{\frac{1}{N}\sum _{i=1}^{N}x_i^2} \end{aligned}$$

Algorithm 1 summarizes the complete workflow for EMG signal preprocessing, feature extraction, and seizure classification.

**Algorithm 1:**
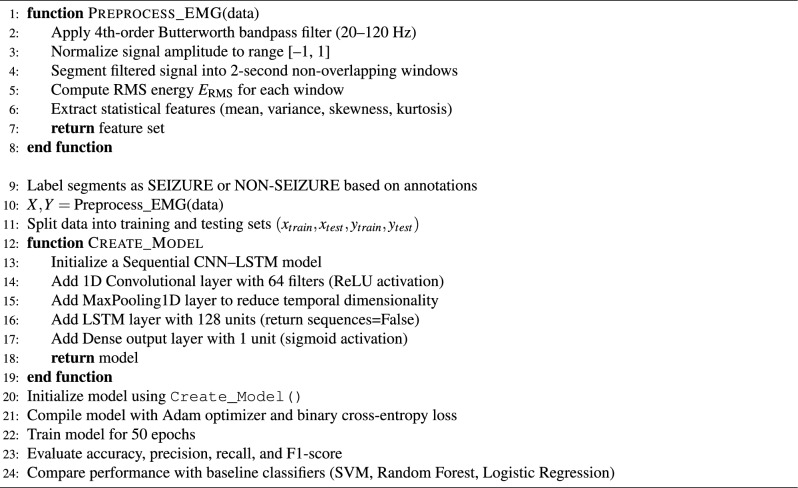
EMG signal processing and seizure detection workflow

Following preprocessing, RMS smoothing was applied and signals were segmented into 2-second windows with a 50% overlap to balance temporal continuity and computational efficiency. From each segment, a multi-domain feature set was extracted, including amplitude-based descriptors (peak-to-peak magnitude, RMS energy), spectral power derived from FFT analysis in the 3–8 Hz seizure-associated band, signal entropy, burst recurrence interval (BRI), and zero-crossing rate (ZCR). These features jointly characterize the rhythmic and chaotic patterns of convulsive muscle bursts.

A Random Forest classifier with 100 estimators achieved an accuracy of 91.2%, precision of 90.8%, recall of 88.4%, and an F1-score of 89.6%, demonstrating strong generalization performance even in the presence of movement artifacts.

**Fig. 19:**
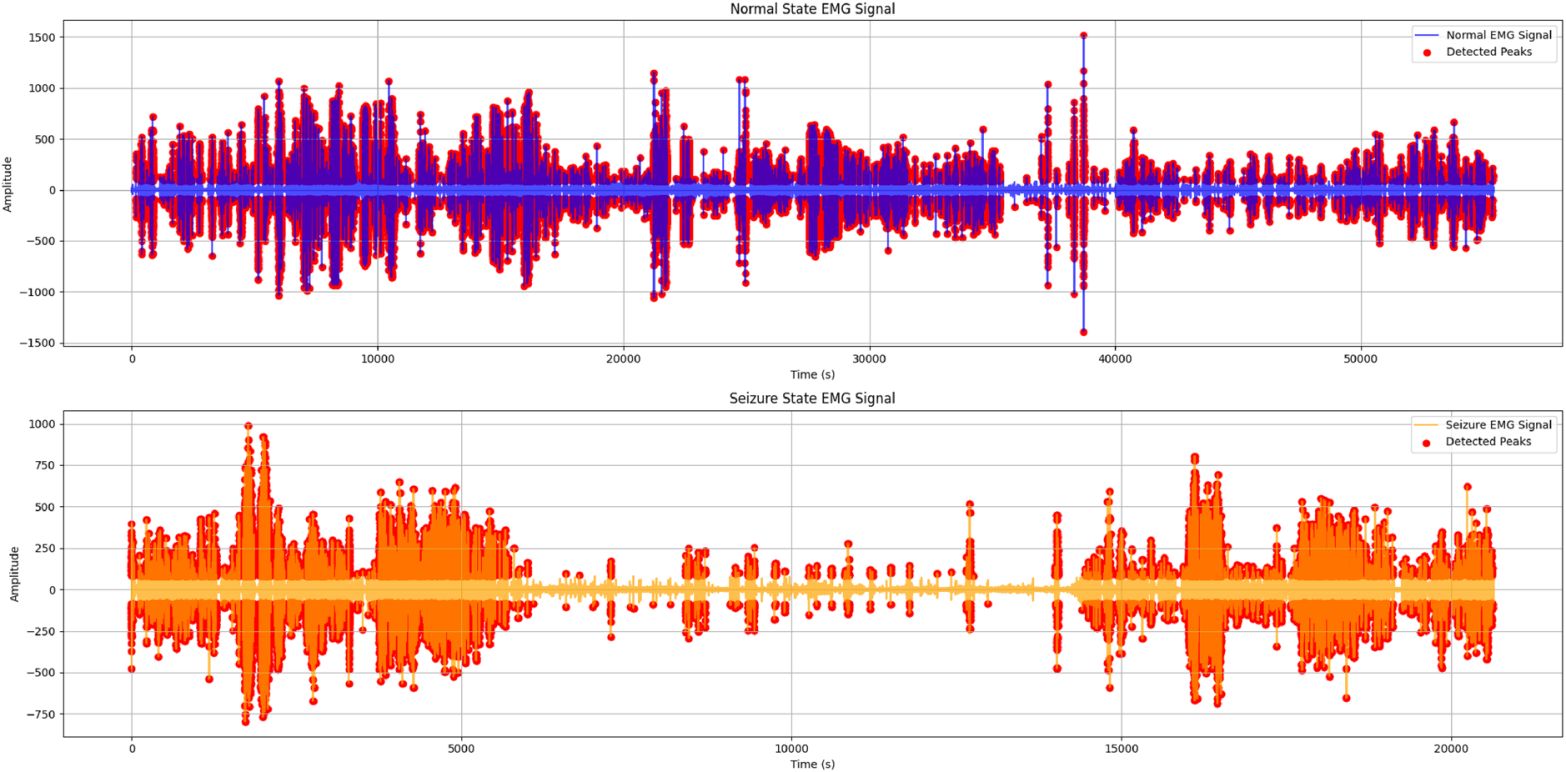
Comparative EMG waveforms for normal, epileptic, and non-epileptic seizure episodes. Epileptic segments exhibit high-amplitude, repetitive burst patterns distinct from baseline muscle activity.

As illustrated in Fig. 19, EMG waveforms corresponding to seizure events exhibit distinct differences in amplitude, periodicity, and burst structure compared to normal muscle activity. While EMG does not replace EEG for clinical diagnosis, it provides a fast, compact, and effective first-line screening mechanism within the **ESC.AI** platform, enabling rapid detection and edge-based alerting for enhanced safety monitoring during school transportation.

#### Contextualization with large-scale ML-based seizure detection studies

Recent large-scale evidence further supports the effectiveness of machine learning approaches for automated seizure detection^51–53^. conducted a comprehensive systematic review and meta-analysis covering 60 studies and 93 datasets, evaluating the diagnostic performance of ML models for epileptic seizure detection using EEG signals.

The meta-analysis reported pooled sensitivity and specificity values of 0.96 and 0.97, respectively, with an area under the curve (AUC) of 0.99, demonstrating the strong potential of both traditional and deep learning models for seizure detection. Subgroup analyses highlighted that model architecture, preprocessing strategies, and dataset characteristics significantly influence detection performance.

While the majority of reviewed studies focused on EEG-based clinical settings, the findings provide strong validation for the use of machine learning in seizure detection tasks. In comparison, the ESC.AI framework adopts a complementary EMG-based approach optimized for real-time, non-invasive screening in transportation environments, prioritizing low latency, robustness to motion artifacts, and edge deployment feasibility. This positions ESC.AI as a practical extension of clinically validated ML methodologies into real-world safety-critical applications.

### Using EMG to find changes in posture

The **ESC.AI** platform incorporates an EMG-based posture change detection subsystem to assess student discomfort and physical strain during school transportation. The module relies on surface electromyography (sEMG) analysis to capture physiological responses associated with prolonged sitting, uneven leaning, thermal stress, and early fatigue manifestations commonly observed in bus environments. By analyzing peak dynamics, signal variability, and amplitude modulation, the system provides insight into postural adaptation under real-world transit conditions.

#### Preprocessing and signal conditioning

Raw sEMG signals were normalized, gap-corrected, and filtered using a 20–40 Hz Butterworth bandpass filter to emphasize tonic muscle activity while suppressing movement artifacts and motor noise. Min–max normalization was applied to ensure consistent feature scaling. This frequency range specifically targets low-frequency muscle contractions associated with static posture maintenance. All preprocessing steps were executed on embedded edge-processing modules to support real-time analysis.

#### Detecting postural events

Dynamic peak detection^54^ was applied to rectified and conditioned signals to identify posture transitions. Extracted physiological features included peak intensity, posture-change frequency, RMS drift, and inter-window activity variation. To evaluate robustness under realistic discomfort scenarios, synthetic EMG stress patterns characterized by reduced inter-peak intervals and elevated background activity were introduced during testing. These controlled perturbations enabled assessment under short-duration posture shifts, asymmetric torso loading, and repeated micro-contraction bursts that serve as ergonomic indicators.

#### Model training and evaluation

A Random Forest classifier with 100 trees and a maximum depth of 5 was trained using inter-burst variance, mean amplitude, peak rate, and peak intensity as primary features^55^. Using an 80/20 split, the model achieved an overall F1-score of 0.93 (Table 6). Although the evaluation dataset was limited in size, the results demonstrate proof-of-concept feasibility for EMG-based ergonomic assessment. Larger and more diverse datasets—particularly those involving seated trunk musculature—are required to enhance ecological validity and demographic generalization.

**Table 6:** Classification performance of the Random Forest model for posture change detection.

Class	Precision	Recall	F1-score	Support
Abnormal	0.95	0.92	0.93	9
Normal	0.90	0.96	0.93	3
**Accuracy**	0.93			
**Macro Avg**	0.93	0.94	0.94	12
**Weighted Avg**	0.93	0.93	0.93	12

#### Signal characterization

**Fig. 20:**
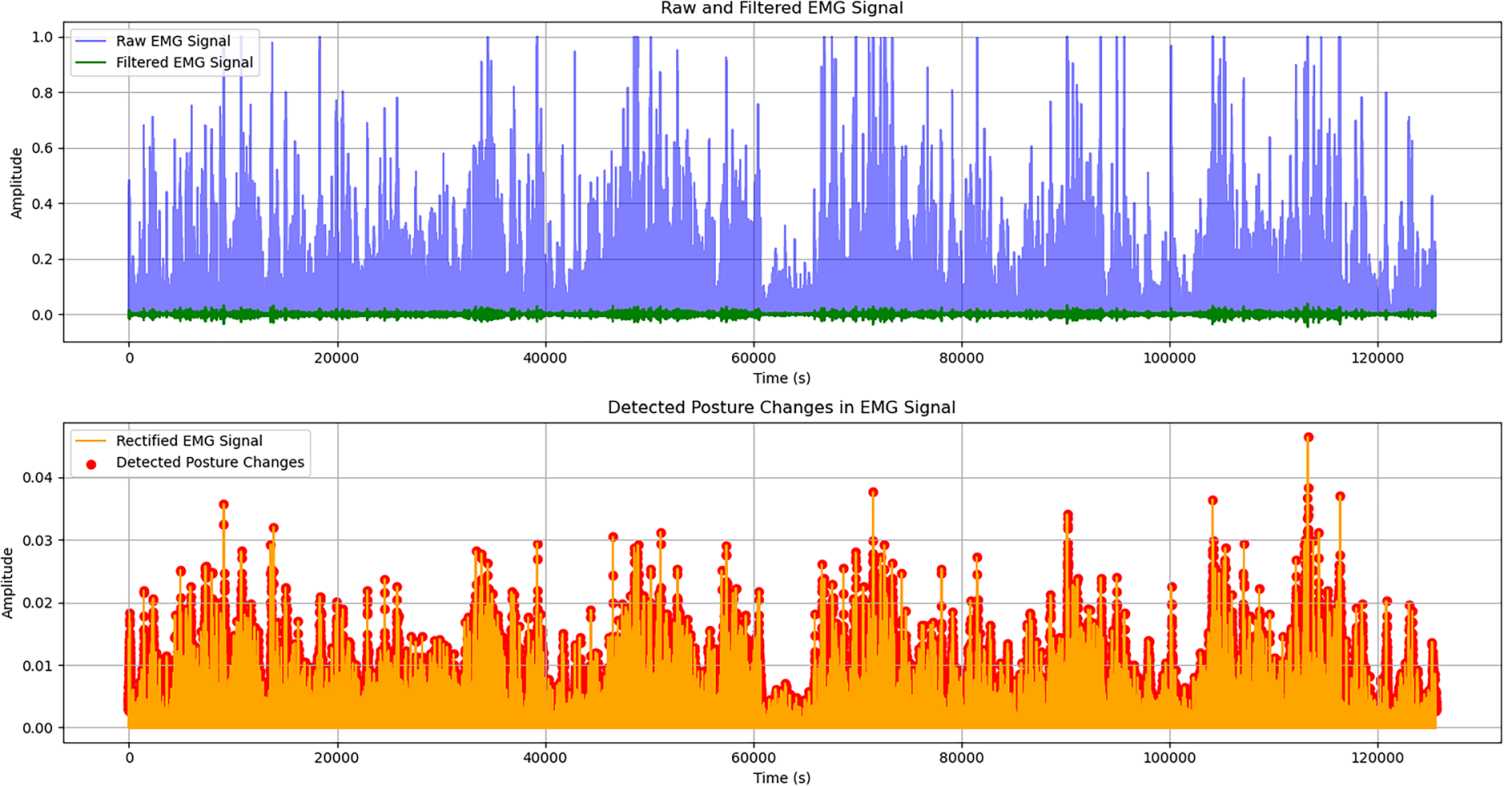
Comparison of raw and bandpass-filtered sEMG signals. Peak detection markers along the rectified signal indicate identified posture changes.

Figure 20 illustrates representative raw and bandpass-filtered sEMG traces annotated with detected posture transitions along the rectified signal path. The system’s ability to distinguish muscle activation from noise demonstrates sensitivity to subtle ergonomic changes in confined bus seating environments. Such detection capabilities support future adaptive interventions, including smart seating adjustments or automated ventilation control responsive to real-time discomfort profiles.

### Driver monitoring via EMG signal analysis

To assess the feasibility of monitoring driver physiological preparedness and neuromuscular stability, **ESC.AI** integrates an exploratory module based on surface electromyography (sEMG) analysis. The module evaluates muscle activation patterns associated with steering control, response latency, and fatigue accumulation during driving-related activities, providing complementary physiological insights beyond vision-based monitoring approaches^56^.

EMG signals were analyzed using 250 ms sliding windows with 50% overlap to extract key physiological markers. Root Mean Square (RMS) and Mean Absolute Value (MAV) were employed to quantify muscle activation intensity, serving as amplitude-based indicators of contraction strength and motor engagement. Reaction time was estimated by measuring the latency between synthetic stimulus events and the onset of EMG activity, defined as the first sample exceeding three times the baseline noise level. This metric provides an estimate of neuromotor responsiveness under simulated driving conditions.

Sudden and involuntary movements were detected by identifying derivative spikes exceeding three standard deviations above baseline activity. Such events may indicate fatigue-induced spasms, loss of motor control, or neuromuscular instability. All features were computed using an edge-optimized analytics pipeline to support future in-vehicle deployment and real-time assessment.

**Fig. 21:**
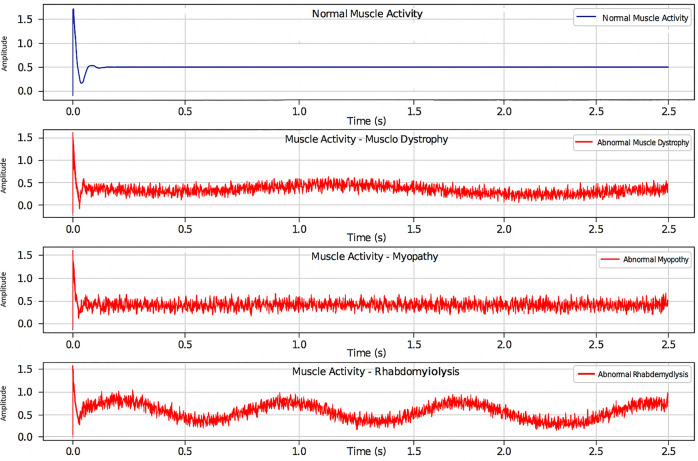
Comparative EMG signal profiles illustrating differences between normal muscle activation and synthetically compromised neuromuscular states. The upper traces show stable voluntary contractions with consistent amplitude and burst timing, while the lower traces demonstrate attenuated amplitude, irregular bursts, and morphological distortion characteristic of neuromuscular impairment.

Figure 21 presents a comparison between normal EMG activity and synthetically degraded signal profiles that emulate neuromuscular impairment. The compromised profiles exhibit amplitude attenuation, irregular burst spacing, and distorted waveform morphology—characteristics commonly associated with myopathy or motor neuron dysfunction. This preliminary evaluation suggests that EMG-based indicators can enhance driver monitoring by revealing subtle physiological degradations not readily detectable through visual cues alone.

### ECG-based health analysis

The **ESC.AI** system incorporates a comprehensive ECG-based health monitoring module designed to assess stress, arrhythmias, and hypertension risk in pediatric passengers during school transportation. The analysis is performed at the window level to support continuous and real-time physiological monitoring. Algorithm 2 outlines the heart rate variability (HRV)-based stress prediction workflow, including signal preprocessing, feature extraction, supervised model training, and evaluation.

ECG signals were processed using Pan–Tompkins R-peak detection, resampled to a unified sampling rate, and segmented into fixed-length windows to compute HRV indices such as RMSSD, SDNN, and mean RR intervals. These features capture autonomic nervous system activity and are widely used indicators of physiological stress and cardiac irregularities.

**Algorithm 2:**
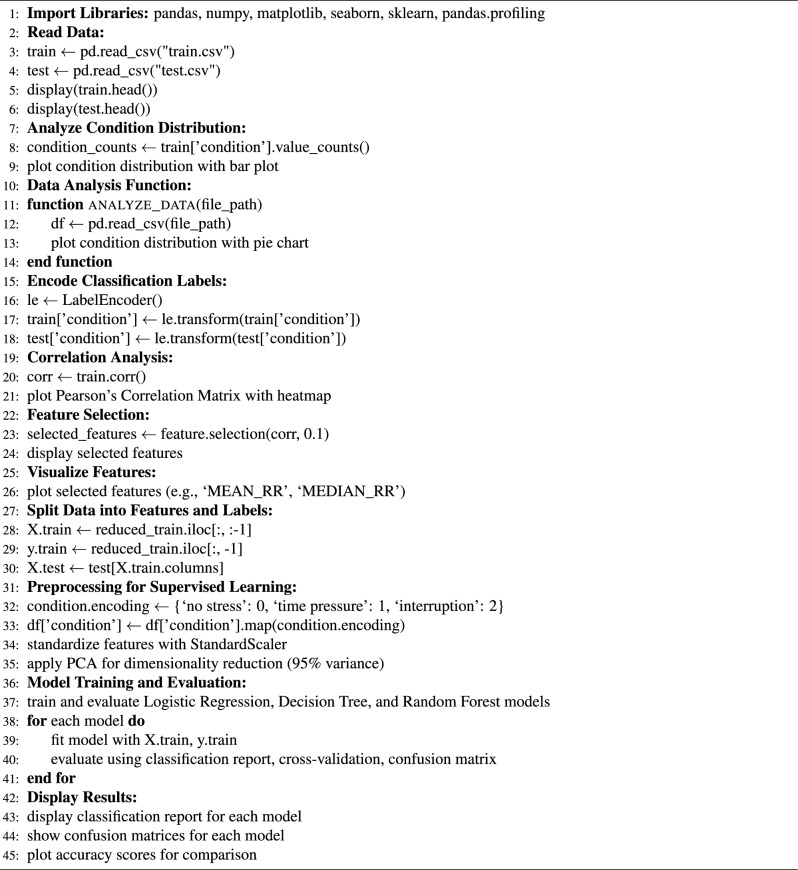
Heart rate variability stress prediction workflow

### Stress detection

Stress identification was performed using heart rate variability (HRV) characteristics to capture autonomic nervous system responses associated with psychological stress. R–R intervals were extracted using the Pan–Tompkins algorithm, and synchronized physiological signals were segmented into 10-second HRV windows for supervised learning.

**Fig. 22:**
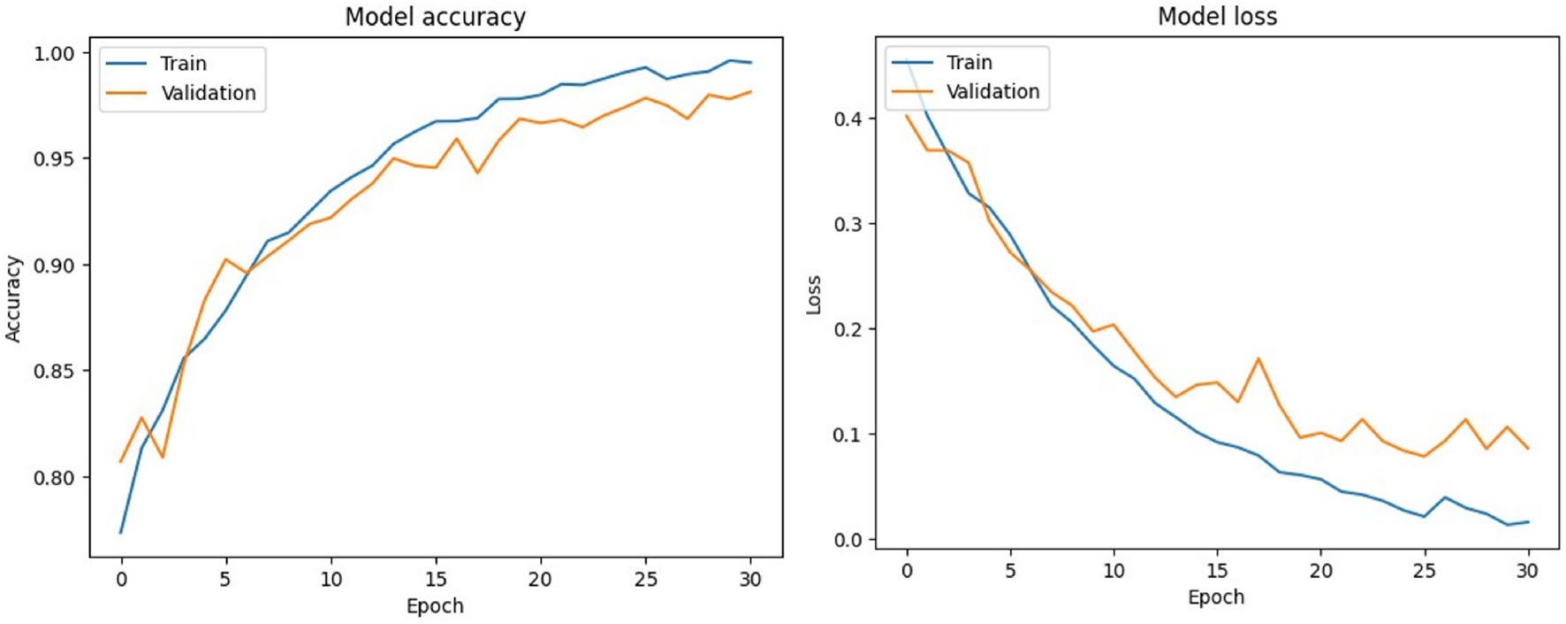
Training and validation accuracy and loss curves for the GRU-based stress classification model.

A gated recurrent unit (GRU) model with two hidden layers (64 units each) was trained using an 80/20 train–test split and cross-entropy loss. The recurrent architecture enabled the model to learn temporal dependencies indicative of autonomic imbalance under stress conditions. The model achieved a test accuracy of 97.8%, an AUC of 0.99, and an F1-score of 96.4% (Fig. 22).

Although the performance was strong, future work should incorporate multi-subject cross-validation and personalized baseline modeling to further reduce overfitting and improve generalization across diverse physiological profiles.

#### Comparison with recent ECG-based stress recognition frameworks

Recent studies have demonstrated remarkable performance in stress recognition from ECG signals using deep learning models. Phukan et al^19^. proposed SONIC, a framework that synergizes multiple vision foundation models to extract rich representations from ECG-derived signals, achieving state-of-the-art performance on the WESAD benchmark with accuracy exceeding 99%.

While SONIC establishes an upper-bound performance under controlled experimental conditions, its reliance on multiple large-scale foundation models results in high computational complexity, making real-time edge deployment challenging. In contrast, the ESC.AI stress monitoring module prioritizes lightweight inference and real-time operation in dynamic transportation environments, where motion noise, sensor variability, and latency constraints are critical factors. Despite operating under significantly more challenging conditions, ESC.AI achieves robust stress detection performance, demonstrating the feasibility of practical, deployable ECG-based monitoring systems beyond laboratory settings.

### Arrhythmia classification

Arrhythmia classification was performed using a one-dimensional convolutional neural network (1D-CNN) designed to learn discriminative morphological patterns from ECG waveforms^57^. ECG beats were segmented based on QRS peak alignment and normalized to fixed-length frames of 180 samples to ensure consistent temporal representation.

The CNN architecture consisted of three convolutional layers with ReLU activation, batch normalization, and dropout regularization to mitigate overfitting. The model achieved a training accuracy of 99.92%, a validation accuracy of 98.09%, and a validation loss of 0.2109, demonstrating strong generalization capability for detecting abnormal cardiac rhythms.

### Prediction of hypertension risk

Hypertension risk prediction was conducted using a Random Forest classifier trained on ECG-derived heart rate variability (HRV) metrics—specifically RMSSD and SDNN—alongside biometric indicators such as age, systolic and diastolic blood pressure, and sleep quality^58,59^.

An RMSSD value below 20 ms was identified as a key marker of autonomic dysfunction and elevated cardiovascular risk in pediatric populations^60,61^. The model achieved an overall accuracy of 0.93, with perfect precision for the High-risk class (1.00) but a lower recall (0.59), indicating that some high-risk cases were misclassified as moderate or low risk.

**Fig. 23:**
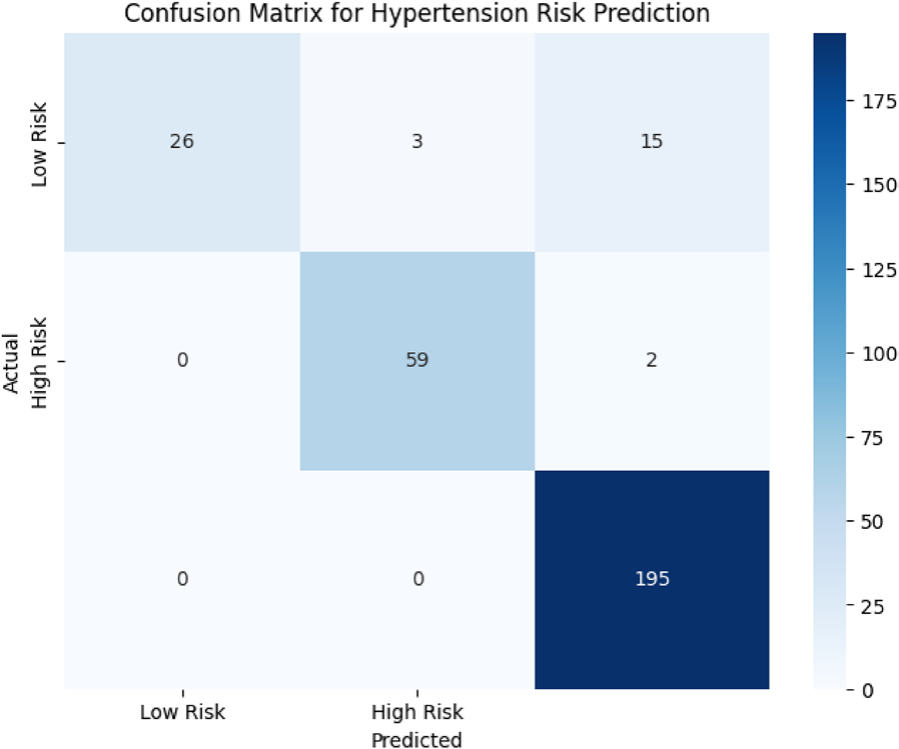
Confusion matrix for hypertension risk prediction showing classification outcomes for low, moderate, and high-risk groups.

This class imbalance is reflected in the confusion matrix shown in Fig. 23, where certain high-risk samples overlap with adjacent categories. Table 7 further reports the detailed classification metrics across all risk categories.

To improve sensitivity for high-risk detection, future iterations should incorporate resampling strategies or weighted loss functions. Overall, these results demonstrate the ability of **ESC.AI** to support real-time cardiac risk monitoring and early intervention, reinforcing its potential for proactive pediatric health surveillance.

**Table 7:** Classification report for hypertension risk prediction.

Class	Precision	Recall	F1-score	Support
High	1.00	0.59	0.74	44
Low	0.95	0.97	0.96	61
Moderate	0.92	1.00	0.96	195
**Accuracy**	0.93			
**Macro Avg**	0.96	0.94	0.85	300
**Weighted Avg**	0.89	0.93	0.93	300

### Results of violence detection

The **ESC.AI** system incorporates a dedicated module for detecting violent behavior to enhance student safety during school transportation. Real-time spatio-temporal video analysis enables the system to identify physical aggression such as striking, shoving, or fighting.

The violence detection module employs an **Inflated 3D ConvNet (I3D)** architecture pretrained on large-scale action recognition data and fine-tuned through transfer learning for in-bus violence scenarios. Fine-tuning was conducted on a curated collection of annotated in-cabin video clips with labels provided by human reviewers.

Input videos were resized to 224$\times$224 pixels and sampled at 30 frames per second. The model was trained for 60 epochs using the Adam optimizer (learning rate = 1e-4), with a dropout rate of 0.5 and L2 regularization applied to mitigate overfitting. Frame-level augmentations, including temporal jitter and horizontal flipping, were employed to enhance generalization.

Evaluation on the validation set demonstrated strong performance (Table 8), despite mild class imbalance. The model achieved an **accuracy of 91.3%**, **precision of 89.6%**, **recall of 90.4%**, and an **F1-score of 90.0%**.

For deployment, inference was performed on an NVIDIA Jetson TX2 edge device, achieving a processing latency of under 3 seconds per video window. This enabled real-time alert generation for supervisors and driver dashboards. All detected incidents were geo-tagged and logged in the student behavioral database for post-event analysis.

These results confirm that the **ESC.AI** violence detection module is both accurate and low-latency, supporting effective in-transit safety monitoring and timely adult intervention.

**Table 8:** Validation metrics for violence detection using the I3D model.

Metric	Value
Accuracy	91.3%
Precision	89.6%
Recall	90.4%
F1-Score	90.0%

### Fall detection results

The **ESC.AI** fall detection module was developed to identify and respond to accidental falls occurring within the constrained environment of a school bus. A fall was operationally defined as a sudden loss of vertical posture characterized by rapid hip-to-floor descent, reduced center-of-mass height, and absence of skeletal recovery within two seconds.

Given crowded seating conditions and frequent occlusions, a pose-based approach was adopted. The detection pipeline employed **OpenPose** for real-time skeletal keypoint extraction, followed by a two-layer **LSTM classifier** (128 units per layer) trained on temporal sequences of 90 frames (approximately 3 seconds at 30 fps).

Input features included joint displacement vectors, hip and head velocity, and torso curvature—targeting instability indicators such as asymmetric limb motion and downward momentum (Table 9). Joint displacement ($\Delta d_t$) and velocity ($v_t$) were computed as:

4 $$\begin{aligned} \Delta d_t = \Vert J_t - J_{t-1} \Vert _2, \qquad v_t = \frac{\Delta d_t}{\Delta t} \end{aligned}$$

Torso curvature ($\kappa _t$) was derived using shoulder, spine, and hip keypoints:

5 $$\begin{aligned} \kappa _t = \frac{\Vert (J_{\text {hip}} - J_{\text {spine}}) \times (J_{\text {shoulder}} - J_{\text {spine}}) \Vert }{\Vert J_{\text {hip}} - J_{\text {spine}}\Vert \, \Vert J_{\text {shoulder}} - J_{\text {spine}}\Vert } \end{aligned}$$

Temporal dependencies across frames were modeled using gated recurrence:

6 $$\begin{aligned} h_t = f(W_h h_{t-1} + W_x x_t + b) \end{aligned}$$

where $h_t$ denotes the hidden state, $x_t$ the input feature vector, and $f(\cdot )$ the activation function.

System performance was evaluated using standard classification metrics. The module achieved an **accuracy of 94.1%**, **precision of 93.5%**, **recall of 92.8%**, and an **F1-score of 93.1%**. The average end-to-end detection latency was approximately **2.2 seconds** on an NVIDIA Jetson Nano, including skeletal extraction and temporal classification.

False positives were primarily attributed to abrupt seatbelt adjustments or intentional crouching motions. Upon fall detection, the system triggers immediate alerts to the driver dashboard and designated staff, including a visual snapshot, timestamp, and geolocation metadata, while logging events in the student safety record.

These results demonstrate the robustness of the ESC.AI fall detection module in distinguishing accidental falls from benign postural movements under real-world bus conditions. Future work will focus on improving resilience to motion blur, crowding, and partial occlusions to support large-scale deployment.

**Table 9:** Validation metrics for pose-based fall detection using OpenPose + LSTM.

Metric	Value
Accuracy	94.1%
Precision	93.5%
Recall	92.8%
F1-Score	93.1%

**Table 10:** Summary of performance metrics across ESC.AI subsystems.

Subsystem	Accuracy	Precision	Recall	F1-score
Driver Monitoring (YOLOv8)	–	89.4%	87.2%	–
Environmental Monitoring	93.0%	86.0%	81.0%	83.0%
Respiratory Analysis	88.7%	–	–	88.7%
Seizure Detection (EMG)	91.2%	90.8%	88.4%	89.6%
Posture Detection (EMG)	93.0%	93.0%	94.0%	93.0%
Stress Detection (ECG)	97.8%	–	–	96.4%
Violence Detection	91.3%	89.6%	90.4%	90.0%
Fall Detection	94.1%	93.5%	92.8%	93.1%
Biometric Authentication	92.0%	92.0%	90.0%	91.0%

This part shows how well the **ESC.AI** Driver Monitoring System (DMS) works, and then it goes on to other evaluations of subsystems in the next sections. The DMS was made with a YOLOv8 model that was trained just for this purpose — to identify five high-risk driving behaviors: open eye, closed eye, cigarette usage, phone use, and seatbelt status. Each of these is very important as signs of tiredness, distraction, or not following safety rules in school transportation settings^62,63^.

The dataset was made up of 5,000 labeled photos (1,000 for each class) that were put together from publicly available driver-behavior sources and added to include video clips taken inside the cabin. Images were normalized and augmented, and their resolution was set to 640$\times$640 (horizontal flips, brightness jitter) to make it more resistant to variations in light and partial obstructions^64^. YOLOv8 was set up for real-time edge deployment with a batch size of 16, a starting learning rate of 0.01 with cosine decay, and customizable anchor boxes that fit the shapes of eyes, portable gadgets, and seatbelt portions. The model was trained for 100 epochs using the Adam optimizer.

Model performance was evaluated using standard object detection metrics. The trained DMS achieved a precision of 89.4%, recall of 87.2%, and an mAP@0.5 of 92.1%. Performance at higher localization stringency yielded an mAP@[0.5:0.95] of 67.6%.

Class-wise analysis indicated strong detection performance for mobile phone usage and seatbelt compliance. Reduced performance at higher IoU thresholds was primarily observed for eye-state classification under low-light or partially occluded conditions, particularly when distinguishing between open and closed eyes^65,66^. These results highlight the inherent challenge of fine-grained facial feature detection in constrained in-cabin environments.

### Cybersecurity module layers

The **ESC.AI** system adopts a multi-layered cybersecurity framework designed to protect sensitive information—including student health metrics, GPS data, behavioral analytics, and operational parameters—by ensuring confidentiality, integrity, and availability across the IoT-enabled school transportation network.

At its core, the system leverages **Public Key Infrastructure (PKI)**, which employs asymmetric cryptography using paired public and private keys for encryption, decryption, and digital signature verification. PKI facilitates device authentication, ensures data integrity, and establishes secure communication channels without exposing confidential information.

During dataset preparation, data were loaded from .npz files for encryption and signing demonstrations. The process includes RSA key generation, hybrid AES–RSA encryption, digital signing, and signature verification steps, as illustrated in Supplementary Figures S5–S6. These layers collectively form the foundation for secure communication and authenticated data exchange within the **ESC.AI** ecosystem.

Algorithm 3 presents the complete workflow for data protection, encompassing key generation, hybrid AES–RSA encryption, digital signing, and signature verification. Each stage ensures the confidentiality and authenticity of transmitted information, enabling end-to-end security across all system modules.

**Algorithm 3:**
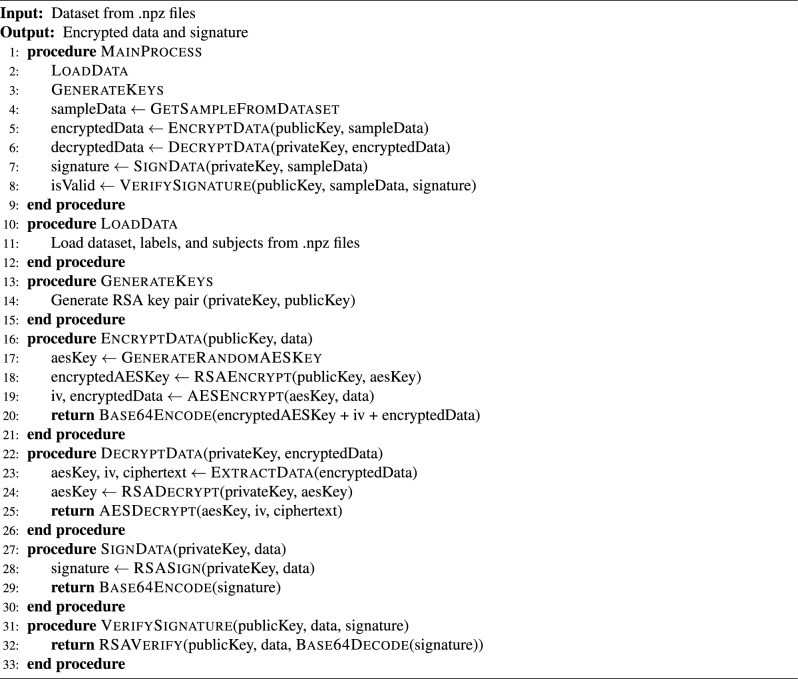
Data encryption and signature verification

Hybrid encryption combines the speed of AES symmetric encryption with the security of RSA asymmetric encryption. This ensures that large datasets are processed efficiently while maintaining the ability to detect and verify tampering^67–71^.

The Advanced Encryption Standard (AES) further enhances the security of operational datasets, including GPS coordinates, driver performance metrics, environmental sensor data, and biometric measurements^67,68^. AES applies block-based encryption using Cipher Block Chaining (CBC) mode, with a derived key generated via PBKDF2 and random salt^69^. A Hash-based Message Authentication Code (HMAC) is integrated for tamper detection^26^.

Encrypted datasets, along with initialization vectors, salts, and HMACs, are securely stored in binary format for controlled retrieval. Decryption occurs only at authorized endpoints, such as bus control centers or parent monitoring applications^72^. Real-time updates enable the system to process traffic and routing data securely without compromising privacy or data integrity^41,67^, as demonstrated in Tables II and III and Supplementary Figure S7.

For lightweight cryptography suitable for resource-constrained IoT devices, the SIMECK block cipher is implemented to encrypt geospatial and metadata fields associated with bus routes, providing efficient protection against threats such as GPS spoofing and data manipulation^41^. SIMECK ensures secure real-time communications with minimal computational overhead, enabling seamless integration into existing IoT frameworks^73^.

Radius-based algorithms enhance spatial security and route integrity within the **ESC.AI** framework^70^. Geofencing establishes circular zones around critical areas, monitoring entry and exit events to verify compliance with authorized pathways. Anomaly detection^68^ continuously compares real-time GPS coordinates and sensor readings against predefined thresholds to identify deviations in parameters such as temperature, heart rate, bus speed, CO$_2$ levels, and BAC values.

Algorithm 4 presents the anomaly detection procedure, while Algorithm 5 outlines secure RADIUS-based authentication and access verification^69^. These pseudocode algorithms describe the procedural flow from client request to server-side validation and secure response issuance^72^. The RADIUS protocol is integrated with a lightweight user database and pre-shared secrets to authenticate access securely, supporting anomaly detection and controlled network access for both administrators and IoT endpoints (see Supplementary Figure S8).

**Algorithm 4:**
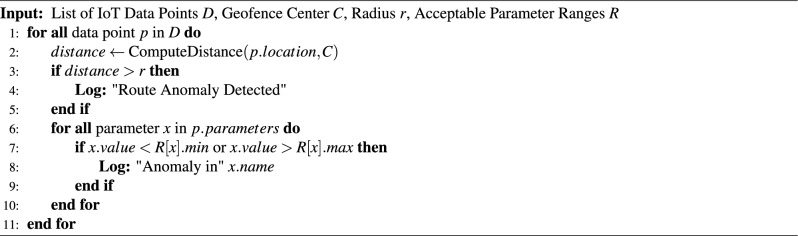
Detect anomalies and route deviations

**Algorithm 5:**
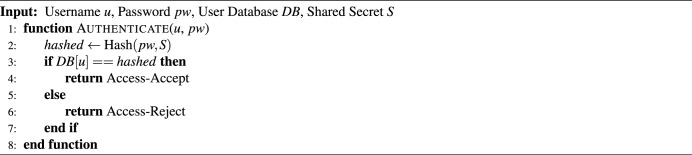
RADIUS authentication handler

Algorithm 5 further explains the stepwise RADIUS authentication process, consisting of five primary stages: user credential input, password hashing with a shared secret, verification against a secure database, conditional authentication decision, and secure response return to the client.

This layered cybersecurity architecture including PKI, AES, SIMECK, and RADIUS protocols ensures that ESC.AI maintains robust confidentiality, integrity, and authentication across all modules, safeguarding sensitive student and operational data while supporting real-time monitoring and control in school bus transportation networks.

### Data visualization on the dashboard

**Fig. 24:**
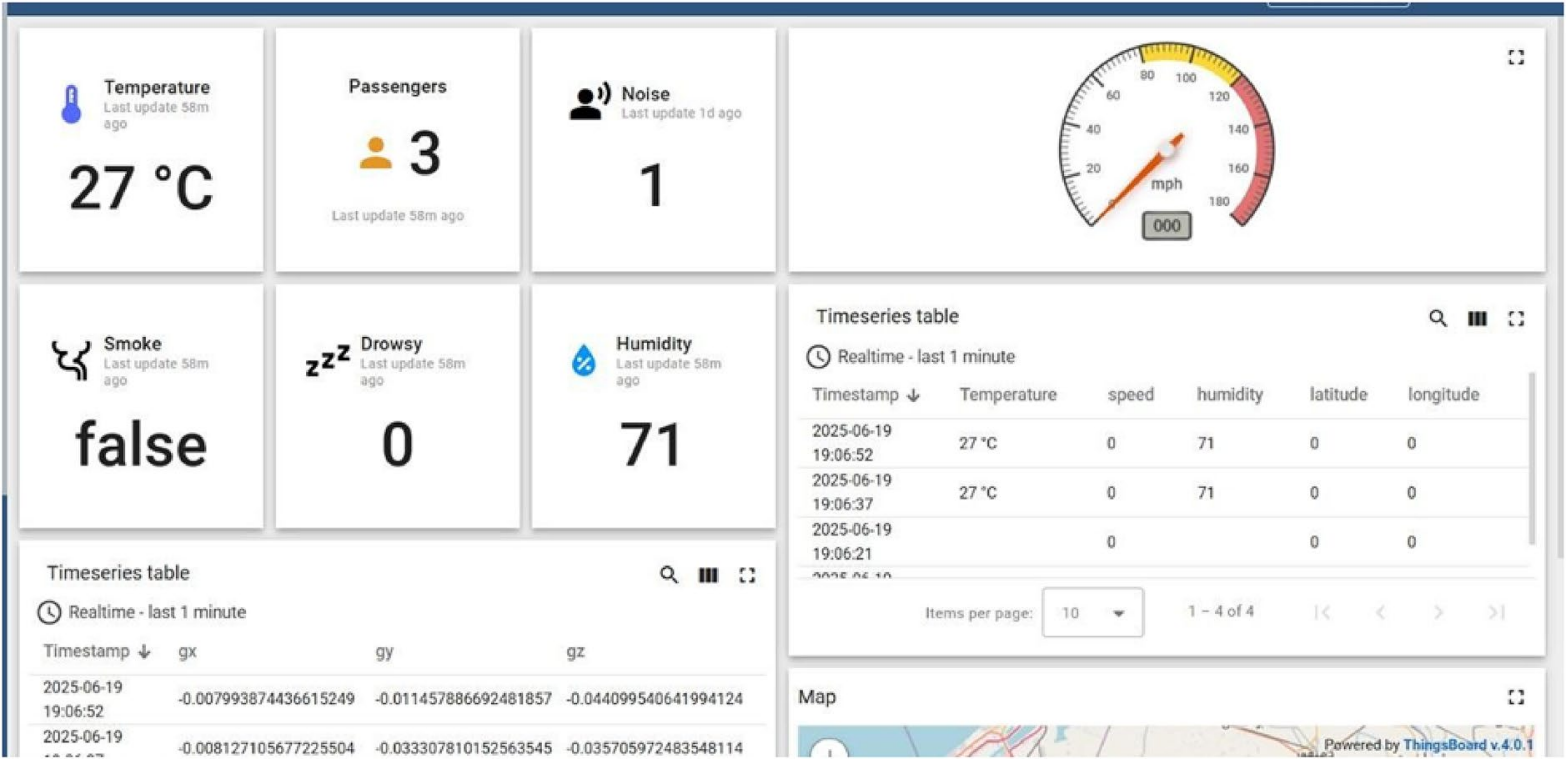
Dashboard interface showing real-time readings for environmental, health, occupancy, and GPS data.

The **ESC.AI** dashboard serves as an all-in-one platform for visualizing real-time environmental and physiological data, ensuring continuous monitoring of every aspect of the school bus environment. Key environmental indicators such as temperature, humidity, gas concentration, and noise levels are constantly measured and classified into three categories—safe, warning, or hazardous—based on predefined safety thresholds (Fig. 24).

At the same time, student health parameters, including heart rate, blood oxygen saturation (SpO_2_), and stress or alertness levels, are displayed to enable early detection of abnormal or risky physiological states during transit^74^.

Biometric authentication data—such as finger impedance values, verification outcomes (authorized or unauthorized), and interdigitated electrode (IDE) contact status—are updated in real time^16^. The dashboard also manages attendance tracking, recording each student’s entry and exit, current bus occupancy, and face recognition outputs distinguishing between known and unknown individuals.

GPS data, live route maps, and GSM network status visualize the bus location and connectivity, while an integrated alerts panel informs supervisors of critical events such as gas leaks, elevated temperatures, low SpO_2_, or door obstructions detected by IR sensors. These combined features create a unified monitoring ecosystem that enhances safety, operational transparency, and rapid incident response.

The supplementary materials provide an in-depth comparison between the **ESC.AI** framework and prior school transportation systems. Supplementary Table S1 reviews existing routing optimization studies, highlighting limitations such as offline computation, lack of real-time updates, and inadequate safety integration. **ESC.AI** addresses these constraints through dynamic GPS-based routing, traffic prediction, geofencing, and student-focused safety mechanisms^7,10,75^.

Supplementary Table S2 compares prior driver monitoring systems that were constrained by single-task objectives, reliance on EEG sensors, or controlled environments^17,76,77^. **ESC.AI** improves upon these by integrating non-invasive camera monitoring to detect multiple behaviors—including drowsiness, mobile phone usage, seatbelt absence, and smoking—while providing real-time alerts and seamless dashboard integration.

Supplementary Table S3 summarizes earlier student health monitoring methods, which were mostly confined to laboratory or clinical settings using static ECG setups^19,58^. In contrast, **ESC.AI** employs wearable ECG and HRV sensors alongside environmental and motion data to achieve continuous real-time health tracking during transit.

Supplementary Table S4 reviews prior biometric authentication technologies that relied on fingerprints, RFID, or laboratory impedance systems, which are often vulnerable to spoofing and lack integration with transportation systems. **ESC.AI** introduces a multi-frequency finger tissue impedance approach with edge-based real-time verification, incorporating liveness detection, student and driver identification, and integration with GPS, routing, environmental, and health modules^15,27,78^.

Finally, Supplementary Figures S5–S8 illustrate the system’s implementation and validation outcomes.

These nn findings emphasize the system’s advances in routing, driver monitoring, health surveillance, data security, and biometric authentication compared with prior solutions.

### Baseline selection and comparison justification

To ensure a fair and meaningful evaluation of the proposed ESC.AI subsystems, baseline models were selected based on three criteria: (i) widespread adoption in related literature, (ii) suitability for real-time or resource-constrained environments, and (iii) representativeness of both classical and deep learning approaches.

For physiological signal analysis tasks (ECG, EMG, and respiratory signals), classical machine learning models such as Support Vector Machines (SVM) and Random Forests were selected as baselines due to their extensive use in biomedical signal classification and their robustness on small- to medium-sized datasets. These models provide a strong non-deep-learning reference point for evaluating the benefits of temporal deep architectures such as CNN–LSTM and GRU-based networks.

For vision-based driver monitoring and in-cabin behavior analysis, YOLOv8 was chosen as the primary baseline due to its state-of-the-art performance in real-time object detection and its favorable accuracy–latency trade-off on embedded edge devices. Unlike two-stage detectors such as Faster R-CNN, which offer higher localization precision at the cost of latency, YOLOv8 enables single-stage inference suitable for safety-critical in-vehicle deployment.

Routing optimization baselines were selected from established formulations of the School Bus Routing Problem using Ant Colony Optimization (ACO) and genetic algorithm-based approaches reported in prior studies. These baselines represent well-known heuristic optimization methods and enable evaluation of ESC.AI under comparable constraints and objective formulations.

Overall, the selected baselines reflect commonly accepted reference methods in the literature and provide a balanced comparison across accuracy, computational complexity, and real-time feasibility. This ensures that observed performance gains arise from architectural and algorithmic contributions rather than from unfair or inappropriate baseline selection.

## Limitations

While **ESC.AI** demonstrates promising technical feasibility and subsystem performance, several limitations must be acknowledged to provide a realistic assessment of the framework:

1. **Dataset Constraints:** The datasets used for training and evaluating driver monitoring, environmental classification, and tissue-impedance biometric authentication were limited in size, diversity, and environmental variability. This restriction may impact generalizability across different student populations, driver behaviors, vehicle types, lighting conditions, and environmental factors. Data augmentation and normalization were applied to mitigate these issues, but larger, heterogeneous datasets are required for broader validation.
2. **Model Assumptions:** The AI/ML models assume consistent sensor placement, reliable signal acquisition, and representative environmental conditions. Variations such as improper sensor positioning, extreme lighting, unexpected passenger behavior, or sudden environmental changes may affect model accuracy.
3. **Potential Biases:** Limited subject diversity and controlled testing conditions could introduce biases in classification outcomes. For example, physiological and behavioral patterns captured during experiments may not fully represent real-world variability among students and drivers, potentially leading to over-optimistic performance metrics.
4. **Situations Where the Model May Fail:**
  Driver behavior detection may degrade under severe occlusion, motion blur, or unanticipated actions.
  Biometric authentication could be affected by excessive finger pressure, extreme skin conditions, or sensor contamination.
  Environmental and physiological risk predictions may fail during rapid, simultaneous events that were not present in the training data.
  Routing optimization relies on accurate traffic and sensor data; sudden road closures or system communication delays could reduce effectiveness.
5. **Limited System-Level Evaluation:** While individual modules were validated experimentally, full end-to-end system performance—including latency accumulation, fault propagation, and resource contention under integrated real-time operation—has not yet been fully assessed.

**Future Work:** Addressing these limitations is a primary goal for future research, including collection of larger, more diverse datasets, real-world deployment in varied operational environments, extended field trials, and comprehensive integration testing to evaluate robustness, scalability, and failure modes.

## Conclusion

This paper presented **ESC.AI**, an integrated cyber–physical framework that combines edge-based artificial intelligence, Internet of Things (IoT) sensing, and adaptive routing principles to support intelligent school transportation monitoring. The proposed architecture unifies multiple heterogeneous subsystems—including driver behavior analysis, environmental condition monitoring, biometric identity verification, and route-awareness mechanisms—within a single cohesive platform designed for real-time, in-vehicle deployment.

The experimental evaluation primarily aimed to validate the feasibility and performance of selected ESC.AI subsystems under controlled experimental conditions. Results from the Driver Monitoring System, implemented using a YOLOv8-based object detection architecture, demonstrated robust recognition of predefined high-risk driving behaviors associated with alertness, distraction, and safety compliance. Environmental monitoring experiments leveraging CO$_2$ concentration and temperature sensing achieved an overall classification accuracy of 93%, indicating reliable discrimination between normal and elevated-risk cabin conditions. Furthermore, the tissue impedance–based biometric authentication module exhibited consistent identity discrimination performance exceeding 90% classification accuracy, highlighting its suitability as a privacy-preserving and spoof-resistant authentication mechanism for transportation environments.

It is important to emphasize that the findings reported in this work demonstrate the *technical viability and functional effectiveness of individual ESC.AI components*, rather than comprehensive system-level safety certification or autonomous decision-making capability. Accordingly, ESC.AI should be regarded as a decision-support and continuous monitoring framework intended to assist drivers, supervisors, and transportation authorities, rather than a fully autonomous or safety-critical control system. Claims regarding direct improvements in operational safety or risk reduction require extended real-world deployment, longitudinal studies, and validation under diverse and uncontrolled operating conditions.

Future work will focus on large-scale field trials across varied geographical, environmental, and operational contexts, alongside deeper investigation of cross-module interactions and system integration effects. Additional efforts will address long-term robustness, scalability, and resilience to real-world variability in traffic density, passenger behavior, and sensor noise. These steps are essential to fully assess the practical applicability of ESC.AI and to support its potential adoption within real-world school transportation systems.

## Supplementary Information

Supplementary Information.

## Data Availability

Data is provided within the manuscript or supplementary information files

## Abbreviations

AI: Artificial Intelligence
ACO: Ant Colony Optimization
AES: Advanced Encryption Standard
API: Application Programming Interface
CNN: Convolutional Neural Network
CO$_2$: Carbon Dioxide
DMS: Driver Monitoring System
ECG: Electrocardiogram
EMG: Electromyography
ESC.AI: Enhanced Safety and Control using Artificial Intelligence
GPS: Global Positioning System
HRV: Heart Rate Variability
IDE: Interdigitated Electrode
IoT: Internet of Things
IR: Infrared
ML: Machine Learning
NDIR: Non-Dispersive Infrared Sensor
NSC: National Safety Council
NHTSA: National Highway Traffic Safety Administration
PERCLOS: Percentage of Eye Closure
RFID: Radio Frequency Identification
RSA: Rivest–Shamir–Adleman (encryption algorithm)
SDNN: Standard Deviation of NN Intervals
TTS: Text-to-Speech
WHO: World Health Organization
YOLOv8: You Only Look Once Version 8

## Declarations

### Declaration on experimental setup

The experiment does not involve human participants and relies solely on commercially available cell line cells(Carboxyl Polystyrene from Spherotech).
