# Supplementary material for: Enhanced smart commuting with artificial intelligence for intelligent health and safety monitoring in school buses
Source: Sci Rep. 2026 Mar 21;16:9665. doi: 10.1038/s41598-026-41628-7 (PMC13009530; doi:10.1038/s41598-026-41628-7)

Supplementary Materials

Supplementary Table S1: Comparative Analysis Of Esc. Ai Against Existing Routing Optimization Studies, Highlighting Methodological Approaches, Limitations Of Prior Work, And Enhancements Introduced By Esc, for School Bus Transportation.

| **Method Used** | **Limitations in Previous Work** | **ESC.AI Improvements** |
| --- | --- | --- |
| Classical route optimization using static GIS and transportation constraints | Traditional GIS-based routing approaches rely on offline computation and predefined schedules, offering limited adaptability to real-time traffic dynamics and safety events. Moreover, these systems do not integrate driver behavior monitoring, student occupancy tracking, or contextual risk assessment, reducing their suitability for safety-critical school transportation applications [1], [2] | Real-time GPS tracking with continuous dynamic re-routing; integration of contextual data including traffic conditions, geofencing, student presence, and driver state to support adaptive and safety-aware route optimization |
| IoT-based traffic prediction with ML forecasting models | Existing IoT and ML-based traffic prediction frameworks mainly focus on traffic flow estimation for urban mobility and smart cities, without addressing school-bus-specific requirements such as child safety, onboard health monitoring, and environmental sensing. These models also lack edge intelligence for low-latency decision-making [3], [4] | Combines IoT traffic prediction with school-bus-specific constraints, multimodal sensing (CO₂, temperature, health signals), and edge AI deployment for real-time, context-aware routing and safety monitoring |
| Multi-objective bus routing (distance, time, cost) using evolutionary optimization | Previous multi-objective optimization methods prioritize operational metrics such as distance, travel time, and fuel cost while overlooking behavioral safety scoring, environmental hazards, and real-time GPS feedback. Additionally, they lack integration with onboard sensing and predictive risk analytics [2], [5] | Introduces safety-weighted multi-objective routing that incorporates crime-risk zones, environmental quality, driver alertness levels, and real-time sensor fusion alongside conventional efficiency objectives |
| Real-time GPS tracking with geospatial analytics for school buses | Prior GPS-based school bus monitoring systems provide real-time location tracking and route visualization but do not include predictive risk analysis, intelligent alerting, or integration with physiological and behavioral sensors. These systems are often reactive rather than proactive in safety management [6], [7] | Enhances traditional GPS tracking through multimodal sensor fusion, predictive congestion modeling, driver monitoring (drowsiness and distraction detection), and closed-loop feedback to the bus system, dashboard, and parent portal |
| Vision-based driver monitoring systems (DMS) | Existing DMS studies primarily focus on drowsiness detection using isolated computer vision models and do not integrate with broader transportation safety frameworks or real-time decision layers. They also lack multimodal fusion with health and environmental data [8], [9] | Integrated edge-based YOLO-driven driver behavior analysis combined with biometric verification, contextual risk scoring, and real-time alert generation within a unified school transportation safety architecture |
| Smart transportation safety systems using cloud-centric AI | Many smart transportation frameworks depend heavily on cloud processing, resulting in higher latency, connectivity dependence, and reduced reliability in mobile environments such as school buses [3], [10] | Edge-centric AI inference enabling low-latency real-time monitoring, offline resilience, secure buffering, and scalable deployment in connectivity-limited transportation scenarios |

[1] Tan, S.-Y.; Yeh, W.-C. The Vehicle Routing Problem: State-of-the-Art Classification and Review. *Appl. Sci.* **2021**, *11*, 10295. https://doi.org/10.3390/app112110295

[2] Chen, A.; Li, T. Modeling and Solving the Multi-Objective Vehicle Routing Problem with Soft and Fuzzy Time Windows. *Systems* **2024**, *12*, 560. https://doi.org/10.3390/systems12120560

[3] Miao, Zhinong & Liao, Qilong. (2025). IoT-Based Traffic Prediction for Smart Cities. IEEE Access. PP. 1-1. 10.1109/ACCESS.2025.3552276.

[4] Rashvand, N.; Hosseini, S.S.; Azarbayjani, M.; Tabkhi, H. Real-Time Bus Departure Prediction Using Neural Networks for Smart IoT Public Bus Transit. *IoT* **2024**, *5*, 650-665. https://doi.org/10.3390/iot5040029

[5] Rizzoli, Andrea-Emilio & Oliverio, F. & Montemanni, Roberto & Gambardella, Luca Maria. (2004). Ant Colony Optimisation for vehicle routing problems: from theory to applications.

[6] Adel, Rana & Hatem, Amira & Sherif, Sameh. (2025). Route Optimization for School Buses Using Real-Time GPS and Geospatial Data. 465-470. 10.1109/ITC-Egypt66095.2025.11186642.

[7] Zakutynskyi, Ihor & Sibruk, Leonid & Kokarieva, Anzhelika. (2023). IoT System for Monitoring and Managing Public Transport Data. WSEAS TRANSACTIONS ON SYSTEMS. 22. 242-248. 10.37394/23202.2023.22.25.

[8] V. S. K. Rupani, V. V. S. Thushar, and K. Tejith, “Real-time drowsiness detection using eye aspect ratio and facial landmark detection,” 2024, doi: 10.48550/arXiv.2408.05836.

[9] Essahraui, S.; Lamaakal, I.; El Hamly, I.; Maleh, Y.; Ouahbi, I.; El Makkaoui, K.; Filali Bouami, M.; Pławiak, P.; Alfarraj, O.; Abd El-Latif, A.A. Real-Time Driver Drowsiness Detection Using Facial Analysis and Machine Learning Techniques. *Sensors* 2025, *25*, 812. https://doi.org/10.3390/s25030812.

[10] Iliopoulou, C., Kepaptsoglou, K. Combining ITS and optimization in public transportation planning: state of the art and future research paths. *Eur. Transp. Res. Rev.* **11**, 27 (2019). https://doi.org/10.1186/s12544-019-0365-5

Supplementary Table S2 Comparative Analysis Of Driver Monitoring Studies Versus The Esc.Ai Framework, Highlighting Limitations In Prior Research And The Comprehensive Improvements Introduced By Esc.Ai In Real-Time Multi-Behavior Detection, Sensor Fusion, And Integration With School Bus Safety Systems.

| **Method Used** | **Limitations in Previous Work** | **ESC.AI Improvements** |
| --- | --- | --- |
| EEG-based drowsiness detection using deep learning with attention mechanisms | Requires wearable EEG headsets that are impractical for real-world public bus deployment; limited scalability; lacks integration with behavioral cues and real-time intervention mechanisms [1], [2], [3] | ESC.AI implements non-invasive vision-based fatigue detection using RGB/IR cameras, integrating eye closure, head pose, and yawning analysis with instant driver alerts and automated event logging |
| Vision-based fatigue and distraction monitoring using CNNs | Detects a limited set of behaviors and is often trained in controlled laboratory environments; lacks robustness in dynamic in-vehicle conditions and does not incorporate multi-sensor fusion [4], [5], [6] | ESC.AI monitors multiple risk behaviors (phone use, smoking, seatbelt compliance, eye state, and distraction) using real-time YOLOv8 with sensor fusion, ensuring robustness in noisy and variable bus environments |
| Deep-learning–based early-warning systems for driver phone usage | Typically single-task models focused only on phone detection; do not address fatigue, smoking, or seatbelt violations; limited alert escalation, logging, and system-level integration [7], [8] | ESC.AI integrates phone detection with fatigue, seatbelt, and smoking recognition into a unified multi-task monitoring pipeline with hierarchical alerting and dashboard integration |
| Computer-vision algorithms for seatbelt detection | Limited to seatbelt classification only; does not evaluate driver attention, fatigue, or distraction behaviors; minimal real-time field validation in transportation settings [9], [10] | ESC.AI provides continuous multi-class behavior recognition with timestamped logs, real-time alerts, and backend analytics dashboard for safety auditing |
| CNN-based driver distraction and behavior detection | Performance degrades under poor illumination and occlusion; absence of physiological or contextual monitoring; limited deployment in real vehicle environments [5], [11], [12] | ESC.AI combines IR and RGB camera streams with contextual AI models for reliable monitoring under both daytime and nighttime conditions |
| Survey and review studies on deep-learning driver monitoring systems (DMS) | Primarily conceptual or review-focused; lack integrated hardware–software implementation and real-world deployment in school transportation ecosystems [6], [13] | ESC.AI delivers a fully implemented multi-sensor driver monitoring system integrated with routing intelligence, student safety modules, and edge–cloud architecture for real-time operation |

[1] Beniczky, S., Wiebe, S., Jeppesen, J., Tatum, W.O., Brazdil, M., Wang, Y., Herman, S.T. and Ryvlin, P. (2021), Automated seizure detection using wearable devices: A clinical practice guideline of the International League Against Epilepsy and the International Federation of Clinical Neurophysiology. Epilepsia, 62: 632-646. <https://doi.org/10.1111/epi.16818>

[2] Djemal, A.; Bouchaala, D.; Fakhfakh, A.; Kanoun, O. Wearable Electromyography Classification of Epileptic Seizures: A Feasibility Study. *Bioengineering* **2023**, *10*, 703. https://doi.org/10.3390/bioengineering10060703

[3] Bai, L.; Litscher, G.; Li, X. Epileptic Seizure Detection Using Machine Learning: A Systematic Review and Meta-Analysis. *Brain Sci.* **2025**, *15*, 634. https://doi.org/10.3390/brainsci15060634

[4] Zhao, Guangzhe & He, Yanqing & Yang, Hanting & Tao, Yong. (2021). Research on fatigue detection based on visual features. IET Image Processing. 16. 10.1049/ipr2.12207.

[5] Essahraui, S.; Lamaakal, I.; El Hamly, I.; Maleh, Y.; Ouahbi, I.; El Makkaoui, K.; Filali Bouami, M.; Pławiak, P.; Alfarraj, O.; Abd El-Latif, A.A. Real-Time Driver Drowsiness Detection Using Facial Analysis and Machine Learning Techniques. *Sensors* **2025**, *25*, 812. https://doi.org/10.3390/s25030812.

[6] J. Wei et al., “A review of YOLO algorithm and its applications in autonomous driving object detection,” *IEEE Access*, vol. 13, pp. 93688–93711, 2025, doi: 10.1109/ACCESS.2025.3573376.

[7] Wang, Z.; Yao, K.; Guo, F. Driver Attention Detection Based on Improved YOLOv5. *Appl. Sci.* **2023**, *13*, 6645. https://doi.org/10.3390/app13116645

[8] V. Rupani, V. V. S. Thushar, and K. Tejith, “Real-time drowsiness detection using eye aspect ratio and facial landmark detection,” arXiv:2408.05836, 2024.

[9] D. Bolya, C. Zhou, F. Xiao and Y. J. Lee, "YOLACT: Real-Time Instance Segmentation," *2019 IEEE/CVF International Conference on Computer Vision (ICCV)*, Seoul, Korea (South), 2019, pp. 9156-9165, doi: 10.1109/ICCV.2019.00925. keywords: {Prototypes;Real-time systems;Image segmentation;Object detection;Detectors;Computational modeling;Task analysis},

[10] Patil, Suvarna & Mujawar, Ayesha & Kharade, Kabir & Kharade, Shraddha & Katkar, Smita & Kamat, Rajanish. (2022). Drowsy Driver Detection Using Opencv And Raspberry Pi3. 19. 6003-6010.

[11] Devi, S. & Anthonysamy, Vinoth. (2025). Real-Time Driver Fatigue Detection and Classification Using YOLOv8-FD: A Precision-Driven Approach for Enhanced Road Safety. Indian Journal Of Science And Technology. 18. 2111-2123. 10.17485/IJST/v18i26.732.

[12] Ye, L.; Wang, D.; Yang, D.; Ma, Z.; Zhang, Q. VELIE: A Vehicle-Based Efficient Low-Light Image Enhancement Method for Intelligent Vehicles. *Sensors* **2024**, *24*, 1345. https://doi.org/10.3390/s24041345

[13] Shaik, T., Tao, X., Higgins, N., Li, L., Gururajan, R., Zhou, X., & Acharya, U. R. (2023). Remote patient monitoring using artificial intelligence: Current state, applications, and challenges. *WIREs Data Mining and Knowledge Discovery*, 13(2), e1485. **<https://doi.org/10.1002/widm.1485>**

Supplementary Table S3: Comparative Analysis Of Student Health Monitoring Studies And The Esc.Ai Framework, Highlighting Prior Limitations And Esc.Ai’s Real-Time, Multimodal, Mobile Deployment Improvements For Continuous Pediatric Monitoring In School Transportation.

| **Method Used** | **Limitations in Previous Work** | **ESC.AI Improvements** |
| --- | --- | --- |
| ECG-based stress classification using foundation models | Primarily evaluated in controlled laboratory datasets with limited motion artifacts; not designed for mobile, noisy, or transit environments; lacks integration with contextual environmental sensing [1], [2], [3] | ESC.AI implements wearable ECG monitoring combined with HRV analysis and environmental sensing (CO₂, temperature, humidity) for real-time stress detection in dynamic school bus environments |
| Multimodal stress recognition using ECG, EDA, and respiration | Validated on benchmark datasets and short-duration recordings; limited real-world deployment and poor robustness to motion noise and edge-device constraints [3], [4], [5] | ESC.AI adapts multimodal physiological monitoring to mobile bus conditions using real-time sensor fusion, edge processing, and automated health alerting |
| Deep-learning digital biomarkers for hypertension using resting ECG | Requires clinical-grade 12-lead ECG and stationary acquisition; lacks continuous and wearable monitoring capability; not suitable for pediatric transit scenarios [6], [7] | ESC.AI employs lightweight wearable ECG sensors with continuous monitoring and risk estimation tailored for pediatric passengers during transit |
| Deep-learning ECG models for early hypertension detection | Focused on adult clinical cohorts; no integration with environmental exposure, motion context, or real-time IoT sensing; limited applicability to cyber-physical transport systems [6], [8], [9] | ESC.AI integrates ECG signals with environmental conditions, motion context, and edge-AI analytics to enable continuous pediatric health monitoring in school transportation systems |

[1] O. C. Phukan, A. Das, A. B. Buduru, and R. Sharma, “SONIC: Synergizing vision foundation models for stress recognition from ECG signals,” arXiv:2404.00827, 2024, doi: 10.48550/arXiv.2404.00827.

[2] Kim, H. G., Cheon, E. J., Bai, D. S., Lee, Y. H., & Koo, B. H. (2018). Stress and Heart Rate Variability: A Meta-Analysis and Review of the Literature. *Psychiatry investigation*, *15*(3), 235–245. https://doi.org/10.30773/pi.2017.08.17

[3] Khan, S.U.; Jan, S.U.; Koo, I. Robust Epileptic Seizure Detection Using Long Short-Term Memory and Feature Fusion of Compressed Time–Frequency EEG Images. *Sensors* **2023**, *23*, 9572. https://doi.org/10.3390/s23239572

[4] A. Gasmi, “Deep learning and health informatics for smart monitoring and diagnosis,” arXiv:2208.03143, 2022, doi: 10.48550/arXiv.2208.03143.

[5] Abdulmalek, S.; Nasir, A.; Jabbar, W.A.; Almuhaya, M.A.M.; Bairagi, A.K.; Khan, M.A.-M.; Kee, S.-H. IoT-Based Healthcare-Monitoring System towards Improving Quality of Life: A Review. Healthcare **2022**, 10, 1993. <https://doi.org/10.3390/healthcare10101993>

[6] Al-Alusi, M.A., Friedman, S.F., Kany, S., Rämö, J.T., Pipilas, D., Singh, P., Reeder, C., Khurshid, S., Pirruccello, J.P., Maddah, M., Ho, J.E., & Ellinor, P.T. (2025). A deep learning digital biomarker to detect hypertension and stratify cardiovascular risk from the electrocardiogram. NPJ Digital Medicine, 8.

[7] Vu T, Petty T, Yakut K, Usman M, Xue W, Haas FM, Hirsh RA and Zhao X (2023) Real-time arrhythmia detection using convolutional neural networks. Front. Big Data 6:1270756. doi: 10.3389/fdata.2023.1270756

[8] Liang, C., Yang, F., Huang, X., Zhang, L., & Wang, Y. (2025). Deep learning assists early-detection of hypertension-mediated heart change on ECG signals. *Hypertension research : official journal of the Japanese Society of Hypertension*, *48*(2), 681–692. https://doi.org/10.1038/s41440-024-01938-7

[9] AReshad, Aquib & Nino, Valentina & Valero, Maria. (2025). Deep Learning-Based Detection of Arrhythmia Using ECG Signals – A Comprehensive Review. Vascular Health and Risk Management. 21. 685-703. 10.2147/VHRM.S508620.

Supplementary Table S4 Comparative analysis of biometric authentication approaches for school transportation, highlighting prior limitations and ESC.AI’s integration of multi-frequency finger tissue impedance with real-time edge verification for enhanced safety and liveness detection.

| **Work / System** | **Biometric Modality** | **Target Context** | **Strengths** | **Limitations Compared to ESC.AI** |
| --- | --- | --- | --- | --- |
| Subramanian, Uma. (2024). Biometric based Students Bus Management System. 8. 167-172. | Fingerprint (optical sensor) | Student boarding and attendance on school buses | Simple concept; uses widely available fingerprint modules; improves over manual attendance | Susceptible to spoofing (gel/silicone); lacks liveness detection; no continuous verification; no integration with health or safety analytics; limited for children’s faint or dirty fingerprints |
| Hoo, Seng & Ibrahim, Haidi. (2019). Biometric-Based Attendance Tracking System for Education Sectors: A Literature Survey on Hardware Requirements. Journal of Sensors. 2019. 1-25. 10.1155/2019/7410478. | Fingerprint + ID/card-based tracking | Student identification and attendance logging | Combines biometric ID with basic tracking; reduces manual errors; low hardware complexity | Focused only on check-in/out; no driver verification; lacks physiological or tissue-level features; vulnerable to lost/stolen cards; no route, environment, or health integration |
| Gadade, Bhanudas & Mulani, Altaf. (2024). IoT Based Smart School Bus and Student Tracking System. 25. 48-53. | RFID tags/cards, GPS, basic sensors | Student tracking and bus location monitoring | Real-time bus location; simple student presence tracking; compatible with legacy fleets | No true biometric authentication; card sharing possible; no spoof-resistance; no stress/health monitoring; limited to location and presence only |

**Supplementary Figure S5.** *Hybrid RSA–AES encryption results demonstrating secure dataset serialization and decryption.*


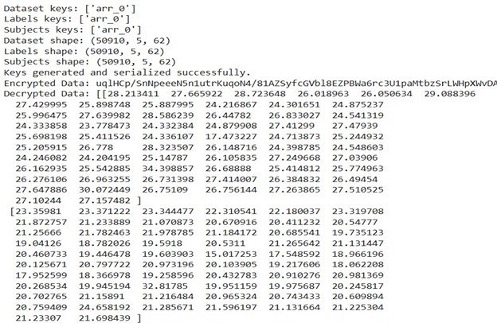


**Supplementary Figure S6.** *Digital signature verification confirming data integrity and authenticity.*


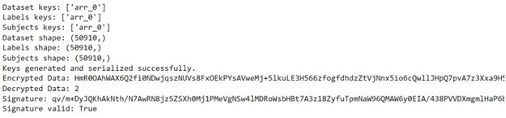


**Supplementary Figure S7.** *AES encryption applied to school-bus operational datasets, ensuring confidentiality of GPS, driver, and fuel information.*


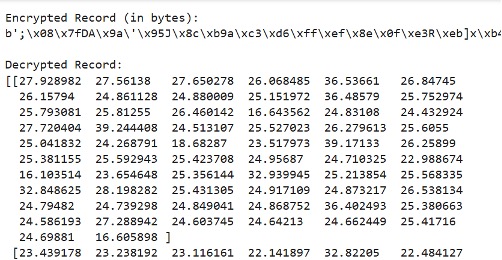


**Supplementary Figure S8.** *Anomaly detection results identifying deviations in temperature, humidity, CO₂, and occupancy readings.*


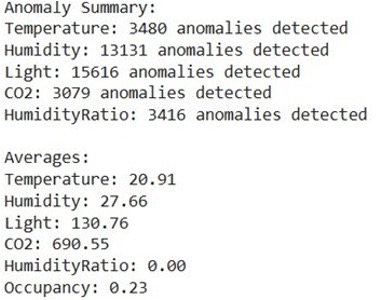

Supplement: Supplementary file 1 — Supplementary Information. [file 41598_2026_41628_MOESM1_ESM.docx]
